# Discovery and technical validation of high-performance methylated DNA markers for the detection of cervical lesions at risk of malignant progression in low- and middle-income countries

**DOI:** 10.1186/s13148-024-01669-z

**Published:** 2024-04-20

**Authors:** Mary Jo Fackler, Madison Pleas, Youran Li, Anushri Soni, Deyin Xing, Leslie Cope, Syed Ali, Quang Van Le, Chu Van Nguyen, Han Thi Pham, Long Minh Duong, Eunice Vanden Berg, Reubina Wadee, Pamela Michelow, Wenlong Carl Chen, Maureen Joffe, Christina Saetan Fjeldbo, Heidi Lyng, Saraswati Sukumar

**Affiliations:** 1 Women’s Malignancies Program, Department of Oncology, Johns Hopkins University School of Medicine, Sidney Kimmel Comprehensive Cancer Center at Johns Hopkins, 1650 Orleans Street, Rm 144, CRB1, Baltimore, MD 21231 USA; 2 Division of Cytopathology, Department of Pathology, Johns Hopkins University School of Medicine, Baltimore, MD USA; 3 Hanoi Medical University, National Cancer Hospital, Hanoi, Vietnam; 4 Department of Quansu Pathology, National Cancer Hospital, Hanoi, Vietnam; 5 Department of Anatomical Pathology, Faculty of Health Sciences, University of the Witwatersrand/National Health Laboratory Service, Johannesburg, South Africa; 6 National Cancer Registry, National Health Laboratory Service, Johannesburg, South Africa; 7 Strengthening Oncology Services Research Unit, Faculty of Health Sciences, University of the Witwatersrand, Johannesburg, South Africa; 8 Department of Radiation Biology, Norwegian Radium Hospital, Oslo University Hospital, Oslo, Norway; 9 Department of Physics, University of Oslo, Oslo, Norway

**Keywords:** Cervical cancer, Quantitative, Assay, Methylation, Markers, Tissue, Cervical smear, Sensitive, Specific, QM-MSP

## Abstract

**Background:**

Cervical cancer remains a leading cause of death, particularly in developing countries. WHO screening guidelines recommend human papilloma virus (HPV) detection as a means to identify women at risk of developing cervical cancer. While HPV testing identifies those at risk, it does not specifically distinguish individuals with neoplasia. We investigated whether a quantitative molecular test that measures methylated DNA markers could identify high-risk lesions in the cervix with accuracy.

**Results:**

Marker discovery was performed in TCGA-CESC Infinium Methylation 450 K Array database and verified in three other public datasets. The panel was technically validated using Quantitative Multiplex-Methylation-Specific PCR in tissue sections (*N* = 252) and cervical smears (*N* = 244) from the USA, South Africa, and Vietnam. The gene panel consisted of *FMN2*, *EDNRB*, *ZNF671*, *TBXT*, and *MOS*. Cervical tissue samples from all three countries showed highly significant differential methylation in squamous cell carcinoma (SCC) with a sensitivity of 100% [95% CI 74.12–100.00], and specificity of 91% [95% CI 62.26–99.53] to 96% [95% CI 79.01–99.78], and receiver operating characteristic area under the curve (ROC AUC) = 1.000 [95% CI 1.00–1.00] compared to benign cervical tissue, and cervical intraepithelial neoplasia 2/3 with sensitivity of 55% [95% CI 37.77–70.84] to 89% [95% CI 67.20–98.03], specificity of 93% [95% CI 84.07–97.38] to 96% [95% CI 79.01–99.78], and a ROC AUC ranging from 0.793 [95% CI 0.68–0.89] to 0.99 [95% CI 0.97–1.00] compared to CIN1. In cervical smears, the marker panel detected SCC with a sensitivity of 87% [95% CI 77.45–92.69], specificity 95% [95% CI 88.64–98.18], and ROC AUC = 0.925 [95% CI 0.878–0.974] compared to normal, and high-grade squamous intraepithelial lesion (HSIL) at a sensitivity of 70% (95% CI 58.11–80.44), specificity of 94% (95% CI 88.30–97.40), and ROC AUC = 0.884 (95% CI 0.822–0.945) compared to low-grade intraepithelial lesion (LSIL)/normal in an analysis of pooled data from the three countries. Similar to HPV-positive, HPV-negative cervical carcinomas were frequently hypermethylated for these markers.

**Conclusions:**

This 5-marker panel detected SCC and HSIL in cervical smears with a high level of sensitivity and specificity. Molecular tests with the ability to rapidly detect high-risk HSIL will lead to timely treatment for those in need and prevent unnecessary procedures in women with low-risk lesions throughout the world. Validation of these markers in prospectively collected cervical smear cells followed by the development of a hypermethylated marker-based cervical cancer detection test is warranted.

**Supplementary Information:**

The online version contains supplementary material available at 10.1186/s13148-024-01669-z.

## Introduction

Cervical cancer ranks as the fourth most diagnosed cancer in the world and a leading cause of cancer death in women throughout the world. The highest burden of cervical cancer incidence is borne by 23 low- and middle-income countries, but the burden of death is largely borne by women in underdeveloped nations such as those in sub-Saharan Africa and Southeast Asia [1]. Women who live in regions of high poverty and immigrants from low-resource countries have twofold higher mortality compared to those with access to screening and treatment [2].

The majority of cervical carcinomas are associated with persistent infection with high-risk HPV strains. Cervical intraepithelial neoplasia (CIN2/3) is a premalignant stage of cervical cancer. CIN3, if left untreated, has as high as 30% risk of progression to invasive cancer within 10 years, [3]. Following therapy, the risk is much smaller, ranging from 1% up to 2.4% among HPV-infected women [4–7]. In contrast to CIN3, the majority of CIN2 regress spontaneously [8, 9]. CIN1, on the other hand, has a low risk of malignant progression. Yet, patients with CIN1 remain under surveillance for up to two years for signs of progression. Higher grades of dysplasia have a shorter time to and greater risk of progression. However, the problem of assigning risk lies in the inability to accurately distinguish between the histological features of the lower grades of dysplasia [10]. Repeated cervical interventions, as is implemented in women with suspicious lesions, has important consequences in young women such as an increased risk of adverse events during pregnancy [11]. In this scenario, less invasive molecular tests that can more accurately detect CIN3+ lesions and provide timely treatment will be useful.

Hypermethylation of DNA is important in silencing of gene transcription and may promote tumorigenesis [12]. Several studies have used DNA methylation markers for detecting cervical cancer [4–7, 13], also comprehensively reviewed in [14]. For example, Brebi et al. identified 2,044 differentially methylated probes in tumor and normal samples, and among these *ZNF516* showed promise with AUCs of 0.76 and 0.92 in two validation cohorts [15]. Building on their own prior work [16], Schmitz et al. developed the GynTect® assay [17] consisting of a panel of 6 markers including ZNF671 which identified 5/5 cancers, 25/162 (15.4%) normal, 12/51 (23.5%) CIN1-2, and 58/88 (66%) CIN3 [17]. The false positive rate for no CIN was 17.2%. The overall sensitivity of the GynTect® assay in women of all ages was shown to be about 67.7% at a specificity of 82.6% [17]. A follow-up paper by the same group on 280 Thin Prep liquid-based cervical scrapes showed similar sensitivity of 64.8% with an improved specificity of 94.8% [18]. A recent paper reported that among 396 cervical smears of women in a colposcopy clinic, the sensitivity and specificity of GynTect® assay for CIN3 detection was 91.2% and 42.2%, respectively, which was a significant improvement over hrHPV detection which showed 95.6% sensitivity and 14.1% specificity in the same study [19]. Another test, the S5 DNA-methylation classifier, based on markers from both host and virus genomes, detected CIN2/3 and cervical carcinoma but not CIN1 or normal cytology [20–23]. This assay measures methylation in 5 different HPV-genes and one host gene, querying a total of 19 CpG sites [21, 24, 25]. Using a S5-methylation classifier cutoff of 3.70 previously demonstrated useful in LMIC settings, Banila et al. recently reported a sensitivity of 62.74% (128/204) for CIN3 and 95.77% (521/544) for cervical cancer at a specificity 74% [26]. The QIAsure Methylation Test, a commercial test analyzing FAM19A4/miR124‐2 methylation, is used as a triage test for women with a positive HPV DNA test or for women with ASC-US cytology to identify those in need of colposcopy. In 2384 HPV‐positive cervical screening samples derived from four EU countries from a cohort of women with no evidence of disease, overall sensitivity for CIN3 detection using QIAsure test was 77% (*n* = 228; 95% CI 71–82), while overall specificity was 78.3% (*n* = 2013; 95% CI 76–80) [27]. The current availability of several large cervical cancer methylome databases provides us the unique opportunity to carefully select markers with increased sensitivity as well as specificity.

Since DNA methylation of key genes occurs early in neoplastic transformation, we hypothesized that a systematic search for methylated markers in public databases will provide a small set of methylated markers that would reliably detect women with concerning cancer and high-grade cervical lesions and clearly distinguish them from women with low-grade lesions and normal cervix. Current worldwide emphasis on screening populations for HPV is well-placed since it identifies those at higher than normal risk of developing cervical cancer. However, HPV testing does not identify those with high-grade intraepithelial lesions (HSIL) or carcinoma who need immediate action. Cervical examination of all HPV-positive women, as currently recommended, consumes time and effort that is ill-afforded by healthcare systems in LMICs. In this study, we describe the use of carefully selected, highly sensitive, and specific hypermethylated DNA markers to triage both HPV-positive and HPV-negative women with HSIL+ cervical disease rapidly and with accuracy.

## Methods

### Sample size

We targeted a sample size of at least *N* = 25 per diagnosis, in each of three countries, the USA, Vietnam, and South Africa, selected to control the precision of the confidence intervals on sensitivity/specificity. Specifically, with sensitivity/specificity above 90%, as we observed in marker discovery, those values can be estimated to within 15% percentage points (based on 90% lower confidence bound).

### Sample collection

Tissues and cervical smears were obtained and tested following approval by The Johns Hopkins Institutional Review Board (Approval No. IRB00241118/CIR00095880), Johns Hopkins Hospital, Baltimore, USA, the Ethics Review Board of National Health Laboratory Services (Approval No. M1911125), Johannesburg, South Africa, and the Ethics Committee of the Vietnam Hanoi Medical University (Approval No. 4400/QD-DHYHN), Hanoi, Vietnam. These sources are heretofore referred to as USA, S. Africa or SA, and Vietnam.

The inclusion criteria used for this study were that the surgically removed tissue samples were from newly diagnosed patients and were histologically confirmed cases of normal/benign, CIN1, CIN2, CIN3, and invasive cancer. Histological confirmation of cytology diagnosis on smears of benign/normal, low-grade intraepithelial lesions (LSIL), HSIL, and invasive cancer was preferred. Clinical history should be available for review, where available. Samples should be from patients more than 18 years of age. Exclusion criterion was that normal/benign samples should not be from women with a history of abnormal PAP smears.

**Fig. 1:**
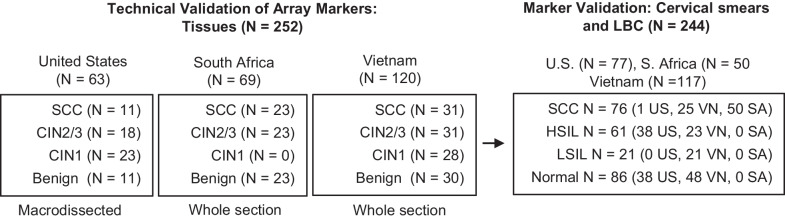
**Samples used for technical validation of the 5-marker panel.** Technical validation of the 5-marker panel was performed using Quantitative Multiplex-Methylation-Specific PCR (QM-MSP) on archival formalin-fixed paraffin-embedded (FFPE)-tissue and cervical smear and liquid-based cytology (LBC) samples. SCC—squamous cell carcinoma; CIN2/3—cervical intraepithelial neoplasia 2/3; CIN1—cervical intraepithelial neoplasia 1; HSIL—high-grade squamous intraepithelial lesion, LSIL—low-grade squamous intraepithelial lesion

Archival unstained formalin-fixed paraffin-embedded (FFPE, *N* = 252) tissue sections and cervical smears (*N* = 244) (Fig. 1) were obtained from women (age > 20 yr) in the USA, S. Africa, and Vietnam who underwent diagnostic cervical procedures for suspicious lesions in the cervix or curative treatment for cervical carcinoma and high-grade squamous intraepithelial lesion (HSIL). All the cervical cell samples from the USA were obtained as liquid-based cytology (LBC); from Vietnam 75% of the samples were cervical smears, while 25% were LBC preparations. All of the cell samples from South Africa were provided as cervical smears. In total, among cell samples from the three countries, 56.6% were cervical smears and 43.3% were LBCs. Histopathology of hematoxylin and eosin-stained tissue sections confirmed the diagnosis, classified as follows: squamous cell carcinoma (SCC), adenocarcinoma (AC) cervical intraepithelial neoplasia grade 3 (CIN3), cervical intraepithelial neoplasia grade 2 (CIN2), cervical intraepithelial neoplasia grade 1 (CIN1), and benign [28]. Three sections from each sample block were obtained from the three institutions for technical evaluation of array markers. Macrodissected or whole sections of cervical tissue samples from a minimal distance of 1 cm from the tumor were used as a source of normal tissue.

Cytology diagnoses on the cervical smear samples were confirmed by histopathology where indicated (abnormal cytology and HPV-16/18 positive, or with severe cervical ectropion), except for cervical smear samples from South Africa for which histopathology was not accessed. Cervical smear cytology classification was performed according to the Bethesda system [29] as follows: squamous cell carcinoma (SCC), high-grade squamous intraepithelial lesion (HSIL), low-grade squamous intraepithelial lesion (LSIL), and negative for intraepithelial lesion (NIEL). Cervical smears (up to 4 slides) were obtained from the three institutions for marker validation. Paired cervical tissue and smears from the same patient were available for a total of 92 cases and controls from Vietnam. Demographic data were collected from the registry or from the medical records at the respective institutions.

### Public datasets used for marker selection and validation

The Cancer Genome Atlas (TCGA) Illumina Infinium HumanMethylation450 BeadChip array (450 K) data collection of Cervical Squamous Cell Carcinoma and Endocervical Adenocarcinoma (TCGA-CESC) consisted of 307 primary cervical carcinomas, 2 metastatic cervical carcinomas, and 3 normal cervical tissues adjacent to tumor. In the TCGA-CESC database, histological types and number of cervical cancers were: primary SCC, *N* = 254, AC, *N* = 53, normal cervical tissue (*N* = 3). Methylation data for normal uterus (N = 45) from TCGA-UCEC (Uterine Corpus Endometrial Carcinoma) database provided additional controls. To validate the selected markers, we used three datasets deposited in the NCBI’s GEO repository. GSE68339 [30] includes DNA methylation profiles from 270 cervical carcinomas, and GSE211668 [31] consists of profiles from 62 cervical carcinomas and 19 normal cervical tissue samples, both on the 450 K array. The third set GSE143752 [32] conducted on an Illumina Infinium 850 K HumanMethylation array (850 K) included 42 CIN3, 40 CIN2, 50 CIN1, 54 normal cervical tissues.

### Genomic DNA extraction, bisulfite conversion, and Quantitative Multiplex-Methylation-Specific PCR (QM-MSP)

Cervical smears (from S. Africa and Vietnam patients) and liquid-based cervical preparations (from USA and Vietnam patients) were processed from a single-stained cytology slide. For tissue analysis, one to two 8-µm unstained sections of formalin-fixed paraffin-embedded sections were used. Following removal of paraffin or the coverslip with xylene, cellular material was scraped with a sterile flat razor blade into 60 µl of digestion buffer [10 mM Tris, 150 mM NaCl, 2 mM EDTA, 0.5% SDS pH 8.5, 100 µg/ml Salmon Sperm DNA (Thermo Fisher Scientific, Carlsbad CA), 2 mg/ml proteinase K (Millipore Sigma, Burlington, MA)]. The sample was incubated at 52^0^ C overnight and then heat-inactivated. DNA conversion was performed using sodium bisulfite according to manufacturer’s directions (EZ DNA Methylation, Zymo Research, Irvine, CA). QM-MSP was performed as previously published [33–35]. A highly detailed step-by-step description of the assay is presented in [33] and summarized below.

The QM-MSP assay is a quantitative nested-methylation-specific PCR method that consists of two PCR reactions: For PCR #1 gene-specific methylation-independent forward/reverse primer sets hybridize to DNA outside the region of interest and co-amplify short fragments of DNA spanning the area of interest. For PCR #2, gene-specific methylation-dependent primer/probe sets for detection of methylated (M) and unmethylated (U) DNA hybridize to amplicons produced in PCR #1. In this step, a two-color real-time PCR method was used to quantitate the relative number of copies of M and U present in a single well of the PCR microplate. The primer and probe sequences for QM-MSP analysis of *FMN2, EDNRB, ZNF671, TBXT,* and *MOS* are presented in Additional file 1: Table S1.

The first Multiplex PCR (PCR #1) used a cocktail of *FMN2*, *EDNRB*, *ZNF671*, *TBXT*, and *MOS* gene-specific external primers to co-amplify 115–225 bp fragments independent of DNA methylation status from a single aliquot of bisulfite-converted DNA, producing amplicons of both unmethylated and/or methylated DNA. All of the sodium bisulfite-converted genomic DNA (10 µL) was amplified in a final volume of 50 µL containing 16.6 mM NH_4_SO_4_, 67 mM Tris pH 8.8, 6.7 mM MgCl_2_, 1.25 mM of dNTP, 10 mM β-mercaptoethanol, 0.1% DMSO, 2 ng of each external primer, and 10 U of Platinum Taq polymerase (Thermo Fisher Scientific). Amplification conditions were 95 °C for 5 min, followed by 35 cycles of 95 °C for 30 s, 56 °C for 45 s, and 72 °C for 45 s, with a final extension of 72 °C for 7 min. A single pair of gene-specific forward/reverse primers produces approximately 10^9^ of copies per µl of DNA amplicons.

In PCR #2, to quantitate the number of M and U amplicons produced in PCR #1, 4 ul of a 1:500 dilution of amplicon DNA from PCR #1 was added to 16 ul of master mix containing 16.6 mM NH_4_SO_4_, 67 mM Tris pH 8.8, 6.7 mM MgCl_2_, 10 mM β-mercaptoethanol, 0.1% DMSO, 300 nM ROX, 200 μM dNTP, 5 ng (final 50 μg/mL) tRNA, 1.25 U Accuris Hot Start Taq DNA Polymerase (Thomas Scientific, Chadds Ford Township, PA, USA), 700 nM each of forward and reverse primer, and 200 nM of each hydrolysis probe in a final reaction volume of 20 ul per well. An ABI 7500 FAST Real-Time PCR System (Applied Biosystems, Foster City, CA, USA) was used for DNA amplification with the following PCR parameters: 95 °C for 10 min, then 40 cycles of 95 °C for 30 s and 65 °C for 1 min. The standard curve (10^–2^–10^–8^ copies of fully methylated cell line DNA), controls (water, unmethylated DNA, fully methylated DNA), and sample DNAs were prepared essentially as described [33].

Based on the cycle threshold, the number of copies was then interpolated from a stock standard curve of serially diluted DNA where U and M copies were set at equimolar concentration (overlapping).

$$\% M = \frac{{\# {\text{copies}}\;{\text{methylated}}\;{\text{DNA}}}}{{{\text{total}}\;\# {\text{copies}}\;{\text{methylated}} + {\text{unmethylated}}\;{\text{DNA}}}} \left( {100} \right)$$

Cumulative methylation (CM) was expressed as the sum of % M for all markers in the 5-marker panel.

### Assay performance criteria

Each plate of real-time PCR #2 contained these controls: (1) multiplexed fully methylated DNA, unmethylated human sperm DNA, and water (no DNA). (2) For any sample, copy number of methylated target gene or unmethylated reference (unmethylated) gene must not be less than Ct (cycle threshold) of 8.0 or exceed the upper end of the standard curve (200,000,000 copies, 10^–2^ dilution of the curve, and for the lower end, the copy number of the unmethylated reference gene must be at least 200 copies, 10^–8^ dilution). (3) The total copies of U + M should be greater than or equal to 20,000 copies to enable detection of at least 200 copies of methylated target. (4) Minimum assay performance criteria to include *R*^2^ ≥ 0.98, efficiency = 90% ± 10%, and slope = − 3.33 ± 10%. (5) Control water, Ct ≥ 38.0. (6) Control fully methylated DNA % M = 100%, and (7) control fully unmethylated human sperm DNA % M = 0%.

### Correlation between methylation and mRNA expression

RNA-seq data in the TCGA-CESC and UCEC databases (described above) were downloaded from Broad Institute of MIT & Harvard (Firehose, https://gdac.broadinstitute.org/)**.** TCGA-CESC, -UCEC 450 K methylation array data and RNA-seq data were compared for each of the five methylation markers.

### Ability of the marker panel to detect HPV-positive and HPV-negative cervical carcinoma

To assess whether the 5-marker panel could detect HPV-positive and HPV-negative cancer, TCGA-CESC and -UCEC array datasets were used. Among the 307 primary cancers, there were 17 HPV-negative tumors. The second, the Genomic Spatial Event (GSE), GSE68339 array dataset [30] was provided by Dr. Lyng and Dr. Fjeldbo (Oslo, Norway), who also provided HPV status information for 270 SCC; among these 20 were HPV-negative tumors.

### Correlation between methylation and age

To estimate the effect of age on DNA methylation levels in our 5-marker panel, we fit linear regression models and calculated Pearson correlation coefficients using β-methylation and age data from normal cervix from TCGA-CESC and normal uterus from TCGA-UCEC.

### Statistical analysis

Database analyses were performed using The Partek® Genomics Suite® software version 6.6 (Partek Inc., Chesterfield, MO) and the R statistical software suite (https://cran.r-project.org/). Figures were generated using GraphPad Prism (GraphPad Software version 10, La Jolla CA). Cumulative stacked histograms and box and whiskers plots were used to display the QM-MSP results expressed as CM-5. Assay performance was reported as areas under the ROC curve as well as sensitivity and specificity. In order to make results more directly comparable, we chose thresholds for distinguishing disease from normal to control sensitivity at 95% and reported the corresponding specificity. Specifically, we calculated the 95th percentile of cumulative methylation in control normal or benign samples for each comparison using the quantile function in GraphPad PRISM as the threshold. ROC metrics were calculated in GraphPad PRISM, which uses a normal approximation by Gagnon [36]. Confidence intervals for sensitivity and specificity are estimated by the Clopper method [37].

## Results

### Study design and workflow

The study design and workflow for discovery and validation of methylation markers is shown in Additional file 1: Fig. S1. First, we conducted in silico analysis of the TCGA-CESC, -UCEC array data collection and identified candidate markers, which we technically validated in external 450 K datasets. Next, we confirmed these findings by testing the selected markers using QM-MSP on tissues and cervical smears from the USA, Vietnam, and S. Africa (Fig. 1). Finally, we evaluated the association between methylation and expression, and methylation and HPV status.

### Marker discovery

**Fig. 2:**
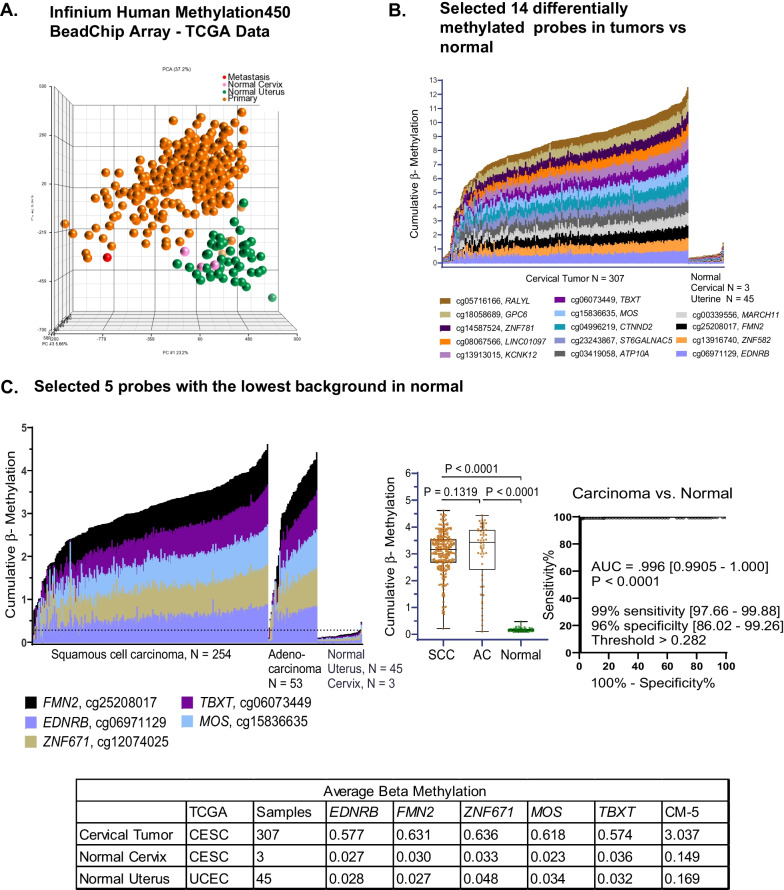
**Marker discovery in The Cancer Genome Atlas-Cervical Squamous Cell Carcinoma and Endocervical Adenocarcinoma (TCGA-CESC) and Uterine Corpus Endometrial Carcinoma (TCGA-UCEC) databases.** **A** Principal component analysis of 485,000 probes shows a clear visual separation of cervical cancer (orange/red) and normal (green/pink) tissue samples. **B**, **C** Histogram plots of cumulative β-methylation in the indicated numbers of carcinomas and normal samples are shown (**B**) for 14 markers and, **C** for the final 5 markers in two histological subtypes of cervical carcinoma squamous cell carcinoma (SCC, N = 254) and adenocarcinoma (AC, N = 53). Also, in (**C**), Mann–Whitney box plots show cumulative β-methylation in SCC and AC samples for the five-marker panel indicating a lower, but not statistically significant difference in methylation (*P* = 0.139) between the two histological subtypes. In the next panel, results of receiver operator curve area under the curve (ROC AUC) analysis are shown. The sensitivity and specificity were based on the 95th percentile of cumulative β-methylation in normal samples (dotted line, histogram). Also tabulated in (**C**) is the average array beta methylation for each gene for TCGA CESC tumor (*N* = 307), CESC normal cervix (*N* = 3) and UCEC normal uterus (*N* = 45). Figure S1 contains additional details of the marker selection process. Tables S2 and S3 provide additional probe information. Abbreviations: SCC, squamous cell carcinoma; AC, adenocarcinoma

The TCGA-CESC and UCEC array datasets were used to interrogate 307 cervical cancers and 48 normal tissues in order to identify markers of cervical carcinoma (Fig. 2). Principal component analysis revealed clear separation between cervical carcinoma and normal tissues (Fig. 2A). For discovery of cervical tumor-specific markers, the array probes were serially filtered in several steps as described in Additional file 1: Fig. S1. The 14 top candidate cervical cancer markers were evaluated as shown in the histogram of cumulative β-methylation in the tumors (Fig. 2B). To arrive at the 5 CpG probes from the top 14 candidate markers selected in this study, 1) we further refined probe selection for achieving a high level of specificity by eliminating the remaining nine probes which were found to have beta methylation values higher than 0.05 units among the 48 normal samples. Data were thus reduced to 5 CpG probes that clearly distinguished SCC and adenocarcinoma from normal tissues as shown in the histogram, boxplots (*P* < 0.0001, Mann–Whitney, and the tabulated average array beta methylation for each gene for TCGA CESC tumor (*N* = 307), CESC normal cervix (*N* = 3), and UCEC normal uterus (*N* = 45) (Fig. 2C). Descriptive statistics for the 5 markers are provided in Additional file 1: Table S2.

In the TCGA databases examined, we observed significant cumulative methylation (CM) levels of the 5-marker panel in squamous cell carcinoma (SCC, *N* = 254) and also in the rarer histological type, adenocarcinomas (AC, *N* = 53) compared to normal tissues in the TCGA-CESC database. Interestingly, CM-5 levels were not significantly different (*P* = 0.1319) between SCC and AC (Fig. 2C). This suggested this marker panel could be broadly useful among different types of cervical carcinoma. The selected probes recognize CpG sites in *FMN2*, *EDNRB*, *ZNF671*, *TBXT*, and *MOS*, as summarized in Additional file 1: Table S2. Additional file 1: Table S3 provides probe index (ID), gene name, location, and function.

The TCGA-CESC methylation profile of the 5-marker panel was examined in three other publicly available datasets (Additional file 1: Fig. S2). The analysis confirmed that the selected markers showed high levels of tumor-specific methylation and low levels of methylation in normal tissues in two databases, GSE68339 [30], and GSE21168 [31]. Intermediate levels of methylation were reported in cervical intraepithelial neoplasia that increased with grade in GSE143752 [32].

### Association between methylation of the five CpG markers and gene expression

CpG methylation can lead to gene silencing and subsequent loss of tumor suppressor function [12]. To determine whether there was a relationship between gain in methylation in the 5-marker panel and loss of gene expression, we plotted TCGA-CESC methylation and RNA-seq expression data for the same CESC cases. Methylation of all 5 CpG markers was significantly higher in tumor than normal (Additional file 1: Fig. S3A). By RNA-seq, *ZNF671*, *EDNRB*, and *FMN2* showed a high level of expression in normal and low expression in tumors. In contrast, gene expression was low in both normal and tumor samples for *TBXT* and *MOS* (Additional file 1: Fig. S3B). Thus, we observed a loss of expression for *ZNF671*, *EDNRB*, and *FMN2*, but not for *TBXT* and *MOS*.

### Analytical validation of 5-marker panel in FFPE tissue from USA, S. Africa and Vietnam

**Fig. 3:**
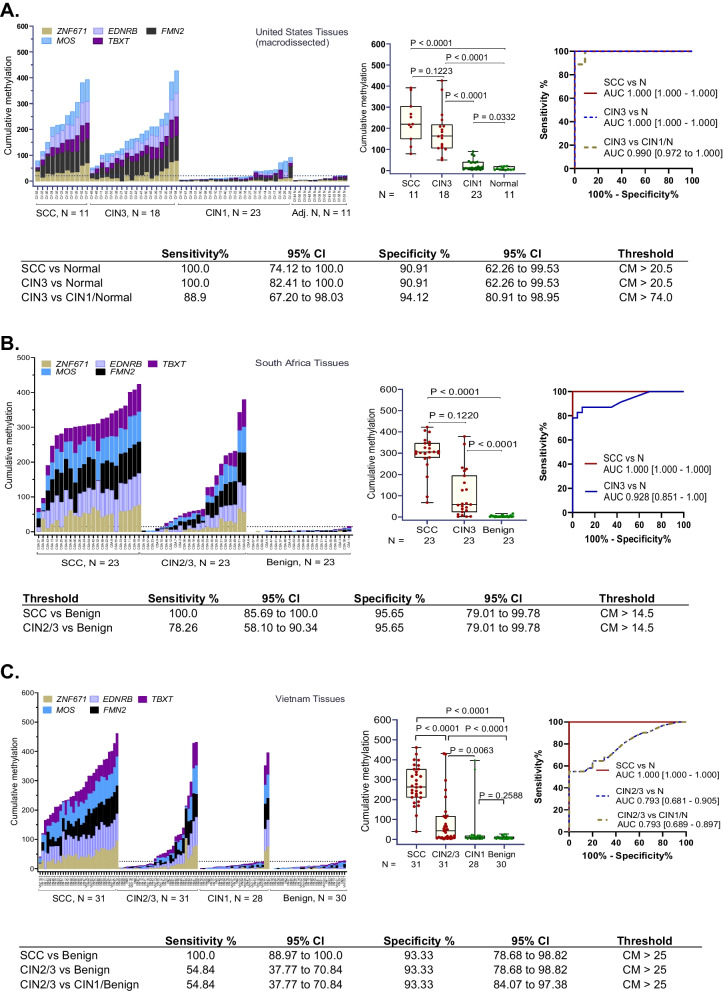
**Technical validation of the 5-marker panel in archival tissue**. Quantitative Multiplex-Methylation-Specific PCR (QM-MSP) was performed on FFPE tissue sections from (**A**) the USA (*N* = 63), **B** S. Africa (*N* = 69), and **C** Vietnam (*N* = 120). The histogram bar indicates the magnitude of Cumulative Methylation-5 (CM-5) (Y-axis) in each sample (X-axis). Box and whisker plots in **A**, **B**, and **C** show comparisons of CM-5 between groups as indicated. Receiver operator curve area under the curve (ROC AUC) results are shown. Sensitivity and specificity were based on the 95th percentile of CM-5 in normal samples in each region (dotted line in histogram). Performance of individual markers from US samples is shown in Fig. S4. SCC—squamous cell carcinoma; CIN2/3—cervical intraepithelial neoplasia 2/3; CIN1—cervical intraepithelial neoplasia 1

To determine whether the 5-marker panel validated by in silico analysis of array datasets shows equally high performance when examined by a laboratory test, we performed analytical validation of the markers using the quantitative methylation-specific PCR method, QM-MSP. This assay was performed on clinical FFPE tissue samples (*N* = 252). The samples from the USA (*N* = 63; Fig. 3A) were macrodissected, while whole tissue sections were used for samples from S. Africa (*N* = 69; Fig. 3B), and Vietnam (N = 120; Fig. 3C). In tissue from USA, cumulative methylation of the 5 markers (CM-5) distinguished between SCC and normal with 100% sensitivity and 91% specificity (ROC AUC = 1.000, 95% CI 1.000–1.000, *P* = 0.0001) and between CIN3 and normal at 100% sensitivity and 91% specificity (ROC AUC = 1.000, 95% CI 1.000–1.000, *P* < 0.0001) (Fig. 3A). Considered individually, the markers achieved significantly higher methylation in both SCC and CIN3 (*P* < 0.0001; Additional file 1: Fig. S4, Additional file 1: Table S4). In tissue from S. Africa, compared to normal, CM-5 detected SCC with 100% sensitivity at 95.65% specificity (ROC AUC = 1.000, 95% CI 1.000–1.000, *P* < 0.0001) and CIN2/3 with 78.26% sensitivity and 95.65% specificity (ROC AUC = 0.928, 95% CI 0.851–1.000, *P* < 0.0001) (Fig. 3B). In tissue from Vietnam, compared to normal, CM-5 detected SCC with 100% sensitivity at 93.33% specificity (ROC AUC = 1.000, 95% CI 1.000–1.000, *P* < 0.0001). Compared to CIN1/normal, CIN2/3 was detected with 54.84% sensitivity at 93.33% specificity (ROC AUC = 0.793, 95% CI 0.689–0.897, *P* = 0.0001). CIN1 methylation was not significantly different from normal (Mann–Whitney *P* = 0.259) (Fig. 3C).

In summary, in cervical tissues obtained from three regions of the world, SCC could be detected in 100% of carcinomas with specificity ranging from 91% to 96%, while CIN3 could be detected with sensitivity ranging from 55% to 89% with a specificity of 91%–96% compared to CIN1/normal. Considered together or separately, in each geographic region, progressively higher methylation occurred as a function of increasing severity of dysplasia.

### Assay validation in cervical smears from USA, S. Africa, and Vietnam

**Fig. 4:**
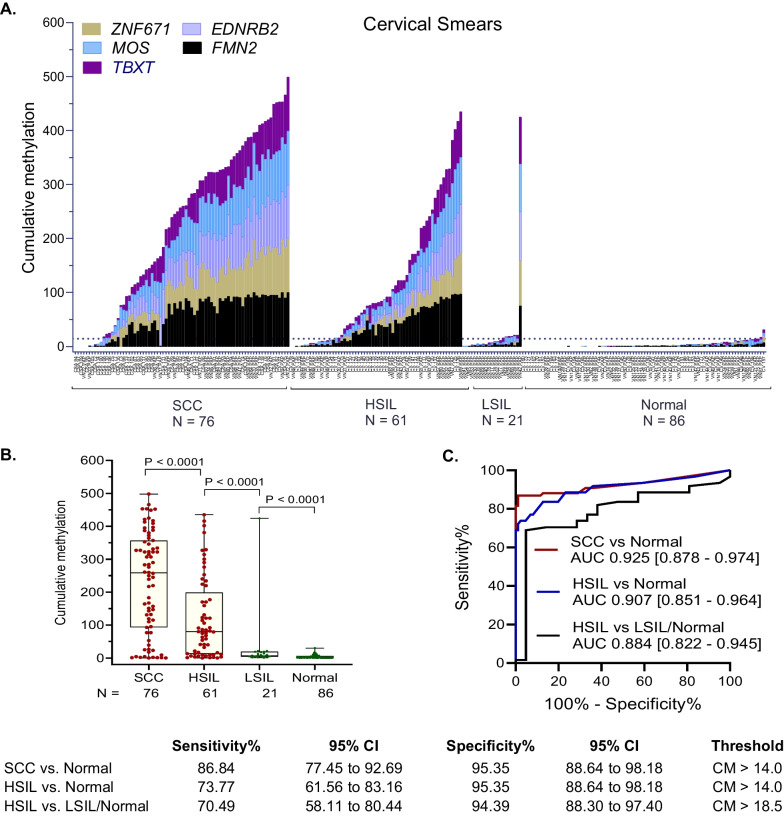
**Detection of cervical cancer and high-grade lesions in cervical smears.** Quantitative Multiplex-Methylation-Specific PCR (QM-MSP) was performed on cervical smears (*N* = 244 total) from the USA (*N* = 77), Vietnam (*N* = 117), and S. Africa (*N* = 50) and data were pooled for analyses. **A** Histogram indicates the magnitude of Cumulative Methylation-5 (CM-5) (Y-axis) for each sample (X-axis). **B** Box and whisker plot shows comparison of CM-5 in samples of cervical smears from normal (N), low-grade squamous intraepithelial lesion (LSIL), high-grade squamous intraepithelial lesion (HSIL), and squamous cell carcinoma (SCC) (*P* < 0.0001, Mann–Whitney). **C** Receiver operator curve area under the curve (ROC AUC) results are shown. Sensitivity and specificity were based on the 95th percentile of CM-5 in normal samples (dotted line in histogram)

Cervical smears are often performed in the screening setting to collect cells from the cervix and vagina for cytological analysis for early detection of precancerous lesions. The potential clinical utility of the 5-marker panel to detect the presence of CIN3 + disease in cervical smears was evaluated by QM-MSP in a total of 244 cervical samples from the USA, Vietnam, and S. Africa. Data from all three countries were pooled to assess CM-5 of SCC, HSIL, LSIL, and normal (Fig. 4). The histogram (Fig. 4A) and box plot (Fig. 4B) showed that CM increased progressively with higher grades of neoplasia. The assay distinguished between normal and SCC with 86.84% sensitivity and 95.35% specificity (ROC AUC = 0.925, 95% CI 0.878–0.974, *P* < 0.0001), and between normal and HSIL with 73.77% sensitivity and 95.35% specificity (ROC AUC = 0.907, 95% CI 0.851–0.964, *P* < 0.0001) (Fig. 4C). Compared to LSIL/normal, HSILs were detected at a sensitivity of 70%, specificity of 94% (ROC AUC = 0.884, 95% CI 0.822–0.945, *P* < 0.0001). Histograms and ROC AUC data of the methylation panel for cervical cell samples are also shown individually for each country (Additional file 1: Fig. S5).

Considered individually, each of the five markers distinguished HSIL from normal in the USA sample set (*N* = 77) (Additional file 1: Fig. S6). As shown in the histograms, cervical smears of HSIL (*N* = 38) showed higher levels of methylation compared to normal (*N* = 38). Individually for each gene, ROC AUC ranged from 0.861 (95% CI 0.776–0.947, *P* < 0.0001) to 0.933 (95% CI 0.875–0.991, *P* < 0. 0001) (Additional file 1: Fig. S6). Thus, each of the five markers sensitively detected HSIL in cervical smears.

### Analysis of the 5-marker panel in paired tissue and cervical smears from the same individuals

**Fig. 5:**
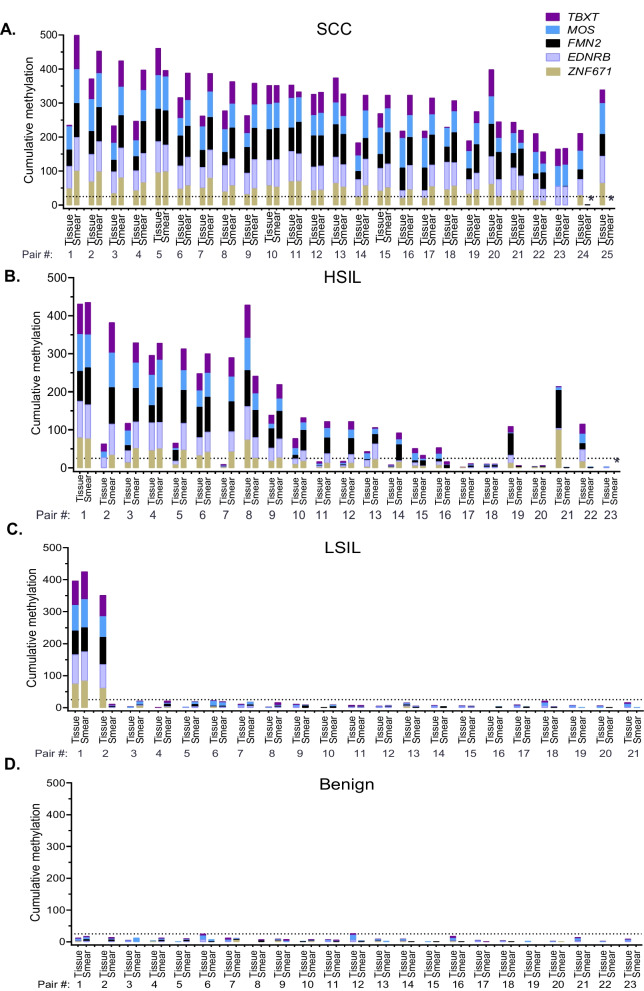
**Paired tissue and cervical smear analysis.** Ninety-two samples of paired tissue (T) and cervical smear/LBC preparations (CS) from Vietnam were tested. Histograms indicate the Cumulative Methylation-5 (CM-5) levels obtained by QM-MSP in patients diagnosed with (**A**) Squamous cell carcinoma (SCC), **B** high-grade squamous intraepithelial lesion (HSIL), **C** low-grade squamous intraepithelial lesion (LSIL), or **D** benign lesion. Data were compiled from samples shown in Fig. 3C and Additional file 1: Fig. S5B

We performed a pairwise comparison between the tissue and cervical smear from the same individual to evaluate whether the methylation results agreed between the sample types. The QM-MSP results of 92 samples from Vietnam were re-analyzed using data presented in Fig. 3C (tissue) and Additional file 1: Fig. S5B (cervical smears). Histogram plots show CM-5 in tissue and cervical smears of patients diagnosed with SCC, HSIL, LSIL, and benign lesions (Fig. 5). There was a high level of agreement in CM-5 methylation between pairs for SCC (23/25) (Fig. 5A), HSIL (17/25) (Fig. 5B), LSIL (20/21, Fig. 5C), and benign (23/23, Fig. 5D). In SCC, discordance was observed in 2 instances where the tissues were positive, while the smear was negative (Fig. 5A). In HSILs, discordance was observed in 4 pairs where smears were positive, while tissues were negative, and in four pairs where tissues were positive, while smears were negative (Fig. 5B). In LSILs, methylation was consistently low in both tissues and smears (19/21) (Fig. 5C). There were two outliers; in one, methylation was very high in both tissue and smear, while in the other, the tissue was positive, while the smear was negative. Strikingly, in all 23 pairs of benign tissues and smears, methylation was below the threshold for normal (Fig. 5D). We concluded that, with few exceptions, cervical smears provided a good reflection of the histopathology of the tissue.

To further clarify the source of samples of tissue, smears, and how many among them were tissue/smear pairs from the same patient, a detailed table of patient samples used in this study is shown (Additional file 1: Table S6). Available demographic data are presented for patient samples from the USA (Additional file 1: Table S7) and Vietnam (Additional file 1: Table S8).

### The 5-marker panel is methylated in both human papilloma virus (HPV)-positive and HPV-negative cervical cancer

**Fig. 6:**
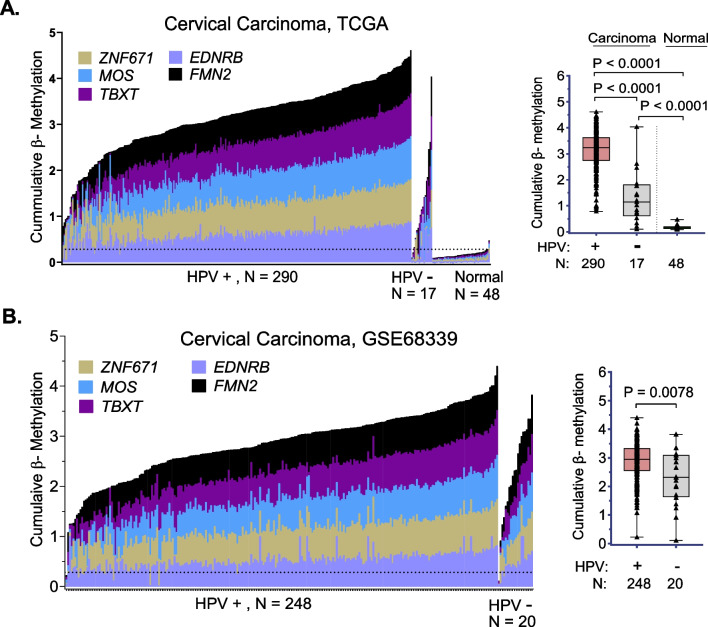
**Human papilloma virus (HPV)-positive and HPV-negative cervical carcinomas are highly methylated for the 5-marker panel**. Histograms and box plots of Cumulative β Methylation in the 5-marker panel in HPV-positive and HPV-negative carcinomas in (**A**). The Cancer Genome Atlas-Cervical Squamous Cell Carcinoma and Endocervical Adenocarcinoma (TCGA-CESC) and Uterine Corpus Endometrial Carcinoma (UCEC) datasets; 17/307 primary tumors were HPV-negative; **B** The Genomic Spatial Event (GSE) GSE68339 database; 20/268 SCC were HPV-negative

The majority of cervical carcinomas are HPV-positive. HPV testing is therefore recommended throughout the world to screen for cervical cancer [38, 39]. Under these circumstances, the 3–10% of carcinomas that are HPV-negative for all the HPV-subtypes currently tested can be missed [40]. To determine whether the 5-marker panel detects cervical carcinoma in both HPV-positive and HPV-negative cases, TCGA-CESC/UCEC and GSE68339 [30] databases were analyzed, correlating β-methylation levels of the 5-CpG marker panel to HPV status (Fig. 6). HPV-negative carcinomas represented 5.5% (17 of 307 cases) in TCGA-CESC (Fig. 6A) and 7.4% (20 of 268 samples) in GSE68339 datasets (Fig. 6B, Additional file 1: Table S8). As observed in the histogram and box plot of the TCGA datasets, HPV-negative samples had significantly higher cumulative β-methylation (*P* < 0.0001) in the 5-marker panel compared to normal cervix and uterus (*N* = 48) In the HPV-negative TCGA-CESC samples, 71% (12/17) of tumors were hypermethylated (Fig. 6A). In the GSE68339 dataset of 268 cancers, where HPV status was determined by a qPCR assay, 95% (19/20) of HPV-negative carcinomas were hypermethylated compared to the normal samples in the TCGA-CESC dataset (Fig. 6B). Interestingly, in both data sets HPV-negative samples were found to have significantly lower methylation than HPV-positive samples (*P* < 0.0001). Although the numbers were small, the results suggested that the 5-marker panel detects both HPV-positive and HPV-negative samples with similar sensitivity.

### Correlation of age to methylation

It is well established that human aging is associated with characteristic changes in DNA methylation throughout the genome [41–43]. To estimate the size of this effect in our markers, we fit linear regression models between age and DNA methylation level (Additional file 1: Fig. S7). In general, we observed that methylation level of our 5-marker signature increased with patient age, although the association did not reach statistical significance (*r* = 0.053 with *P* = 0.604) in the TCGA-UCEC population. Thus, we concluded that CM-5 methylation was not significantly correlated with age in either CESC or UCEC normal datasets.

## Discussion

In this paper, we describe our systematic effort to identify specific CpG dinucleotides that are highly and differentially methylated in cancer but not in normal tissues, and compile and validate a new 5-marker panel for cervical cancer. We also describe the technical validation of this panel of methylated gene markers. Through QM-MSP analysis of the 5-marker panel in nearly 500 histologically confirmed tissues and cervical smears obtained from three countries we show that the test performs with a high level of sensitivity and specificity to detect CIN3+ disease. Although preliminary, to our knowledge the study describes markers that perform with a high level of accuracy.

An inability to achieve optimal sensitivity and specificity using various versions of commercial HPV tests in detecting early precancerous cervical lesions has led to a strategy of combining HPV DNA testing with cytology triage, and other molecular markers [44–47]. Others have tested methylated gene markers alone, or combined with HPV-testing and cytology to determine whether the three-pronged approach would improve detection and risk stratification of low-grade lesions [26]. A number of commercial methylation tests have used single-host gene methylation of POU4F3 (sensitivity 74% and specificity 89%) [48], PAX1 (sensitivity 78%, and specificity 92%) [49], or panels of two, PAX1/ZNF582 (sensitivity 78.85% and specificity 73.55%) [50] to six markers [17–19, 51]. A number of studies have used the 6-marker GynTect® assay. Sensitivity for CIN3 detection in these studies ranged from 64 to 91% and specificity ranged from 42 to 95% [17–19, 51]. The S5-classifier consists of markers of HPV gene methylation combined with a host gene marker, EPB41L3, showed a high sensitivity of 93.2% but a low specificity of 41.8% [20]. Among these, the FAM19A4 and hsa-miR124-2 markers, marketed as the QIAsure methylation test, have been tested more extensively and showed high sensitivity (77%, *N* = 228; 95% CI 71.0–82.0) and specificity (78.3%, *N* = 2012; 95% CI 76.0–80.0) for detection of CIN3 [27]. In pooled data analysis of cervical smears from three different countries and ethnicities, our QM-MSP-based determination of CM-5 in HSIL compared to LSIL/normal samples achieved a sensitivity of 70% (95% CI 58.11–80.44), specificity of 94% (95% CI 88.30 – 97.40), and ROC-AUC = 0.884 (95% CI 0.822–0.945). Although we have not yet validated our markers in uniformly collected samples from a well-planned prospective study, our 5-marker panel for cervical cancer shows strong potential for future testing and development.

Our methylated marker panel consists of five genes, *FMN2*, *EDNRB*, *ZNF671*, *TBXT,* and *MOS*. The genes are potential growth suppressors with varied functions (Additional file 1: Table S3). The products of two of these genes are DNA-binding transcription factors. TBXT has a potential role in promoting cellular transformation and progression through epithelial mesenchymal transition [52]. The zinc finger-containing ZNF671 protein has a metastasis suppressor role through regulating the Notch and Wnt/β-catenin pathways [52, 53]. EDNRB was identified as a G-protein coupled receptor that activates the phosphatidylinositol calcium signaling cascade and is also reported to be aberrantly expressed and differentially methylated in cancer [54, 55]. Another molecule in our panel is MOS, a serine threonine kinase that activates MAPK signaling [56]. MOS has also been implicated in inducing aneuploidy/polyploidy in cancer cells by regulating actin filaments during cell division [57, 58]. FMN2, a member of the Formin family implicated in multiple neurodevelopmental disorders, is an actin-binding protein that regulates actin networks and cell polarity and is essential for meiotic metaphase [59]. While EDNRB and FMN2 as well as TBXT and ZNF671 could have potential tumor suppressor functions, MOS is a well-known oncogene. One approach to test whether the differential methylation at the selected CpG sites is biologically relevant is to query whether methylation of the gene in that CpG-rich region is associated with reduced expression. Examining methylation and expression in the same samples in TCGA-CESC revealed that hypermethylation of three of the genes, *ZNF671*, *EDNRB*, and *FMN2* was associated with loss of gene expression, while *TBXT* and *MOS*, although hypermethylated in tumor, showed essentially non-detectable expression in tumors or normal tissues (Additional file 1: Fig. S3).

Several factors might affect our data. First, both the tissue and cervical smears were samples of convenience obtained from three different countries and as a result, suffered from an uneven distribution of grades and number of specimens in the different cohorts. The tissues examined were uniformly unstained formalin-fixed and paraffin-embedded sections, while the cervical cell samples were not. Sixty percent of cervical cell samples were stained cervical smears, while 40% were liquid-based cytology (LBC) preparations, which might affect cytological diagnoses. Tissues from the USA were macrodissected, while whole sections from South Africa and Vietnam were used. The diagnosis was based on cytology for the cervical smears that was confirmed by histopathology at the collaborating center by the study pathologist, but no central pathology review was performed. Other limiting factors were that HPV status and age information was not available for all cases and controls. We used the laboratory assay, QM-MSP, for the quantitative analysis of the methylated markers. However, this assay needs expertise and a laboratory setup that may not be available in LMICs. A simplified Q-MSP assay or a commercial automated assay would be more suitable and could be developed for use in LMICs.

This is the first report of the 5-marker panel and the results are encouraging. The robust performance of the markers presents a strong rationale for further investigation in a large prospective clinical validation study with an independent sample set, accompanied by accurate HPV-testing, detailed patient characteristics, and centralized cytology and pathology diagnosis. The QM-MSP assay could be a valuable triaging tool for further clinical intervention and for risk stratification in developed countries and could provide higher sensitivity and specificity for detection of cervical neoplasia. The test could be automated and modified for high throughput as demonstrated by our studies using GeneXpert cartridges for detection of tumor-specific methylated DNA in fine needle aspirates of the breast lesion for distinguishing between benign and malignant growths and to assess changes in tumor load in circulating DNA in patients undergoing chemotherapy [26, 60–62]. We are currently developing a cartridge-based liquid biopsy-methylated marker assay for colon cancer detection [63].

## Conclusions

In this study, we have demonstrated the value of a systematic stepwise search for methylated markers focused on the detection of CIN3+ cervical cancer. The markers underwent rigorous technical validation on tissues and cervical smears representing each stage of disease progression. The markers achieved a high sensitivity and specificity of detection of high-risk HSILs and cancer in cervical tissues and smears from three different countries, supporting the possibility of a universally applicable set of markers. Moreover, the marker panel was equally sensitive for the detection of HPV-positive or HPV-negative cancer. If reproduced in large studies, it will result in change of practice, streamline the pathway to biopsy, and result in tremendous savings in healthcare.

### Supplementary Information

**Additional file 1:** Figure S1: Marker discovery workflow.  Figure S2: Validation of TCGA-CESC 5-marker panel using external databases.  Figure S3: Association between CpG methylation and gene expression for individual genes in the 5-marker panel.  Figure S4: Contribution of individual markers of the 5-marker panel to detect cervical neoplasia. Figure S5: Detection of cervical cancer and high-grade lesions in cervical smears from U.S., Vietnam and S. Africa. Figure S6: The 5-marker panel is highly methylated in cervical smears from patients with HSIL and SCC. Figure S7. Association between DNA methylation and age in normal/benign tissue in the TCGA UCEC database. Table S1: Primer/probe sequences for QM-MSP.   Table S2: TCGA-CESC: Descriptive statistics for the 5-marker panel. Table S3: TCGA-CESC Cervical marker ID, gene name, location and function.  Table S4: Descriptive statistics for QM-MSP methylation of individual 5-marker panel in tissue. Table S5: HPV status of patients in dataset GSE68339. Table S6: Detailed description of samples used in this study.  Table S7- US patient characteristics.  Table S8 Vietnam Sample Demographic Data.

## Data Availability

The datasets used and/or analyzed during the current study are available from the corresponding author on reasonable request.
