# Supplementary material for: Discovery and technical validation of high-performance methylated DNA markers for the detection of cervical lesions at risk of malignant progression in low- and middle-income countries
Source: Clin Epigenetics. 2024 Apr 20;16:56. doi: 10.1186/s13148-024-01669-z (PMC11032610; doi:10.1186/s13148-024-01669-z)
Supplement: Supplementary file 1 — Additional file 1: Figure S1: Marker discovery workflow. Figure S2: Validation of TCGA-CESC 5-marker panel using external databases. Figure S3: Association between CpG methylation and gene expression for individual genes in the 5-marker panel. Figure S4: Contribution of individual markers of the 5-marker panel to detect cervical neoplasia. Figure S5: Detection of cervical cancer and high-grade lesions in cervical smears from U.S., Vietnam and S. Africa. Figure S6: The 5-marker panel is highly methylated in cervical smears from patients with HSIL and SCC. Figure S7. Association between DNA methylation and age in normal/benign tissue in the TCGA UCEC database. Table S1: Primer/probe sequences for QM-MSP. Table S2: TCGA-CESC: Descriptive statistics for the 5-marker panel. Table S3: TCGA-CESC Cervical marker ID, gene name, location and function. Table S4: Descriptive statistics for QM-MSP methylation of individual 5-marker panel in tissue. Table S5: HPV status of patients in dataset GSE68339. Table S6: Detailed description of samples used in this study. Table S7- US patient characteristics. Table S8 Vietnam Sample Demographic Data. [file 13148_2024_1669_MOESM1_ESM.pdf]

SUPPLEMENTAL DATA FILES

Discovery and technical validation of high-performance methylated DNA markers for the detection of cervical lesions at risk of malignant progression in low- and middle-income countries

Mary Jo Fackler[1], Madison Pleas[1], Youran Li[1], Anushri Soni[1], Deyin Xing[1], Leslie Cope[1], Syed Ali[2], Quang Van Le[3], Chu Van Nguyen[4], Han Thi Pham[4], Long Minh Duong [4], Eunice Vanden Berg[5], Reubina Wadee[5], Pamela Michelow[5], Wenlong Carl Chen[6,7], Maureen Joffe[7], Christina Saeten Fjeldbo[8], Heidi Lyng[8,9], Saraswati Sukumar[1]*

[1] Department of Oncology, Johns Hopkins University School of Medicine, Baltimore, MD
[2] Division of Cytopathology, Department of Pathology, Johns Hopkins University School of Medicine, Baltimore, MD
[3] Hanoi Medical University, National Cancer Hospital, Hanoi, Vietnam
[4] Department of Quansu Pathology, National Cancer Hospital, Hanoi, Vietnam
[5]Department of Anatomical Pathology, Faculty of Health Sciences, University of the Witwatersrand/National Health Laboratory Service, Johannesburg, South Africa
[6] National Cancer Registry, National Health Laboratory Service, Johannesburg, South Africa
[7] Strengthening Oncology Services Research Unit, Faculty of Health Sciences, University of the Witwatersrand, Johannesburg South Africa
[8] Department of Radiation Biology, Norwegian Radium Hospital, Oslo University Hospital, Oslo, Norway.
[8, 9] Department of Physics, University of Oslo, Oslo, Norway.

Contents:

1. Figure S1: Marker discovery workflow
2. Figure S2: Validation of TCGA-CESC 5-marker panel using external databases
3. Figure S3: Association between CpG methylation and gene expression for individual genes in the 5-marker panel
4. Figure S4: Contribution of individual markers of the 5-marker panel to detect cervical neoplasia
5. Figure S5: Detection of cervical cancer and high-grade lesions in cervical smears from U.S., Vietnam and S. Africa
6. Figure S6: The 5-marker panel is highly methylated in cervical smears from patients with HSIL and SCC.
7. Figure S7. Association between DNA methylation and age in normal/benign tissue in the TCGA UCEC database

8. Table S1: Primer/probe sequences for QM-MSP
9. Table S2: TCGA-CESC: Descriptive statistics for the 5-marker panel
10. Table S3: TCGA-CESC Cervical marker ID, gene name, location and function
11. Table S4: Descriptive statistics for QM-MSP methylation of individual 5-marker panel in tissue
12. Table S5: HPV status of patients in dataset GSE68339
13. Table S6: Detailed description of samples used in this study.
14. Table S7- US patient characteristics
15. Table S8 Vietnam Sample Demographic Data

SUPPLEMENTAL FIGURES

Figure S1

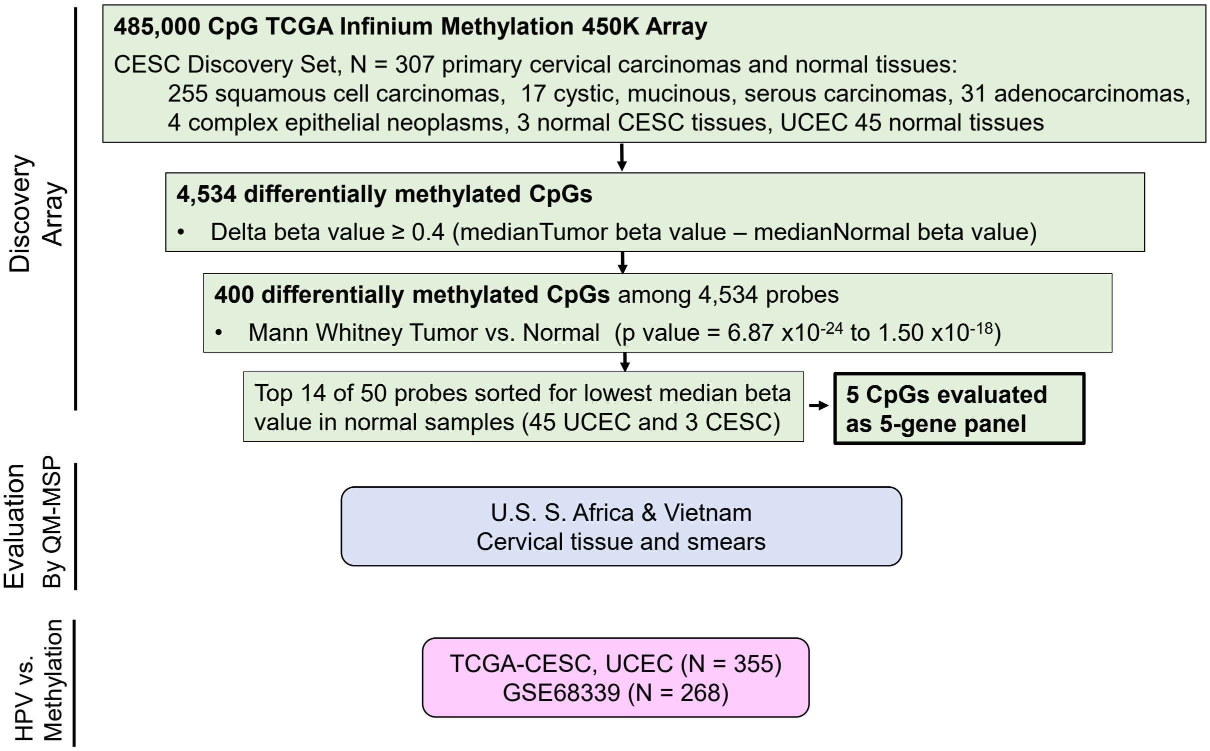

Figure S1. Marker discovery workflow. Arrayed cervical tumor and normal samples from TCGA-CESC and -UCEC were used to identify markers of cervical cancer. These were found to be highly methylated in cancer but not in normal. Among 485,000 total CpG probes, five were selected for further evaluation by QM-MSP in samples from U.S., S. Africa and Vietnam. FFPE tissue samples were used for initial screening of the markers. Cervical smear samples were used to validate the markers. Methylation in HPV-positive and HPV-negative tumors were evaluated in TCGA-CESC and -UCEC, and GSE68339 datasets. Tables S1 and S2 contain specific probe ID information, location, tumor/normal ratio of methylation and functional characteristics of CpG sites in regions represented by the 5-gene panel.

Figure S2

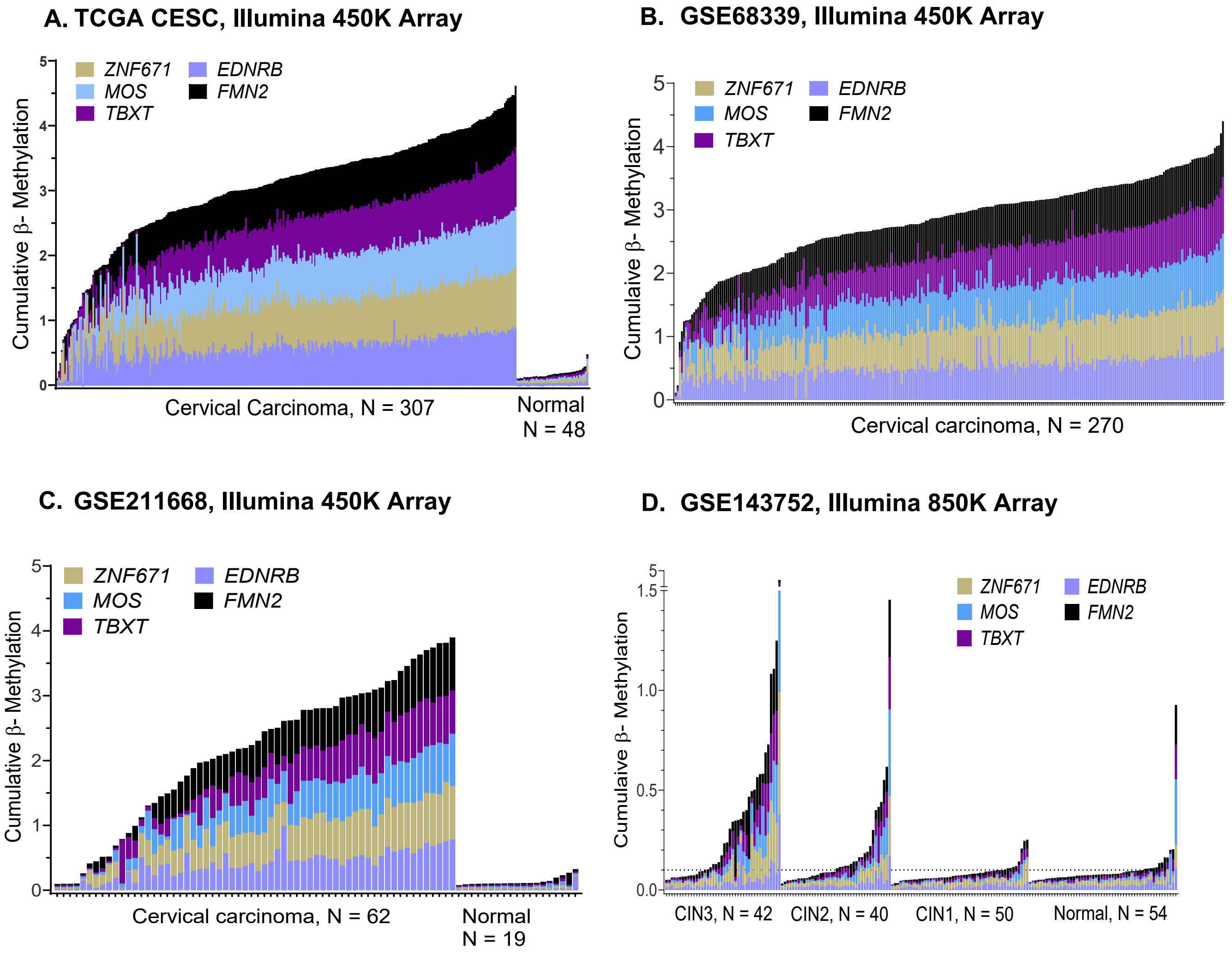

Figure S2. Validation of TCGA-CESC 5-marker panel using external databases. Histograms indicate cumulative β-methylation (Y-axis) for the 5-marker panel in each sample (X-axis). The height of each colored segment represents the intensity of the β-methylation signal in each of the 5 markers in **A.** TCGA-CESC and TCGA-UCEC 450K Illumina array platform used for marker discovery. **B.** GSE68339, **C.** GSE211668 and **D.** GSE143752 were used for validation of TCGA data in A. Figure S1 contains additional details of the marker selection process.

Figure S3

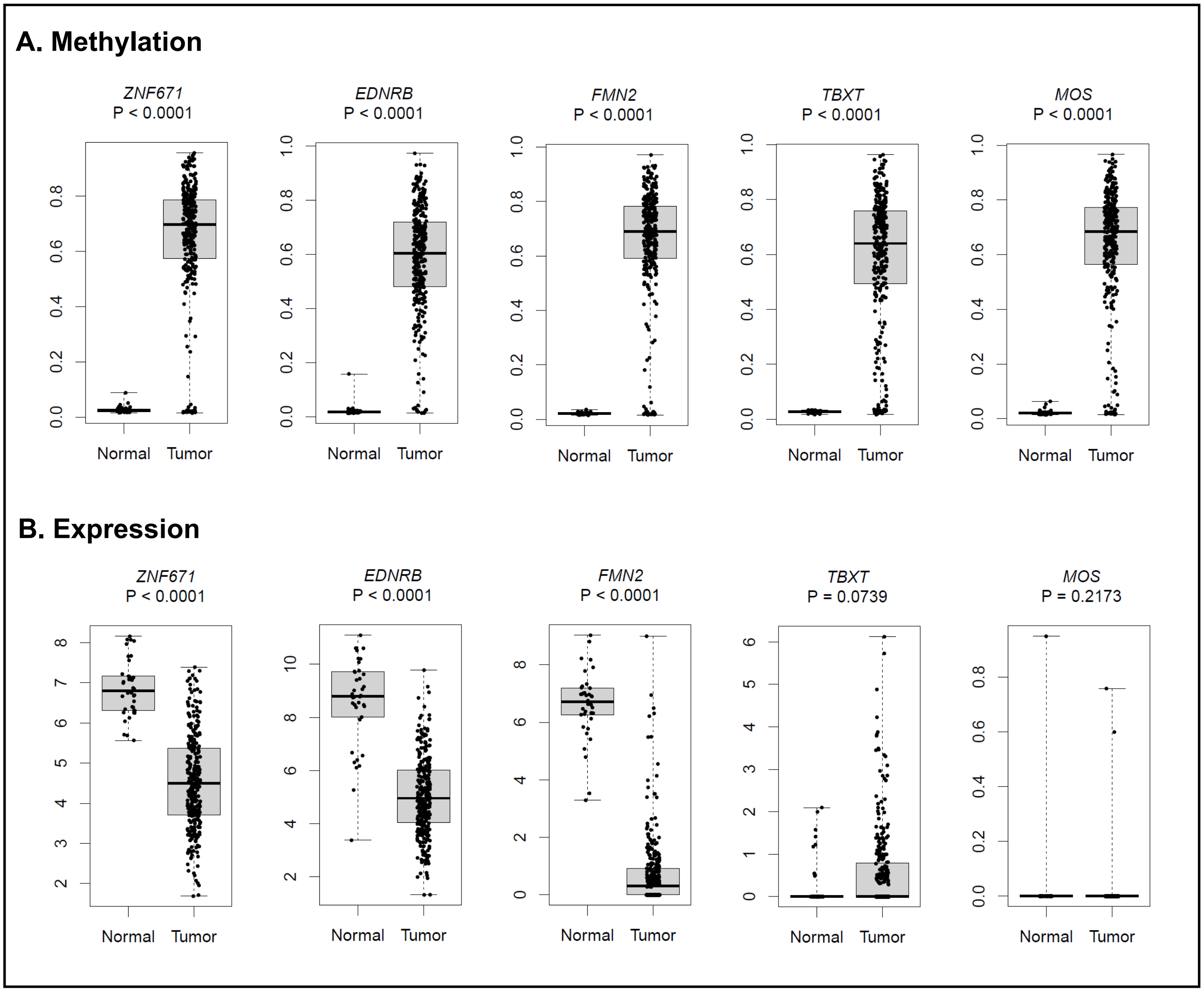

Figure S3. Association between CpG methylation and gene expression for individual markers in the 5-marker panel. TCGA-CESC 450K array data and RNA sequencing data were compared for each of the markers in normal (N = 48, 45 from TCGA-UCEC, 3 -CESC) and tumor (N = 307 from TCGA-CESC). The data are presented as box and whiskers plots with Mann-Whitney statistics. Methylation results indicate significantly higher methylation in tumor compared to normal (P < 0.0001) for all five markers. A relationship between high level of methylation and low expression was observed for *ZNF671*, *EDNRB* and *FMN2*, but high methylation in *TBXT* or *MOS* was not reflected in change of expression which was low in both normal and tumor samples.

Figure S4

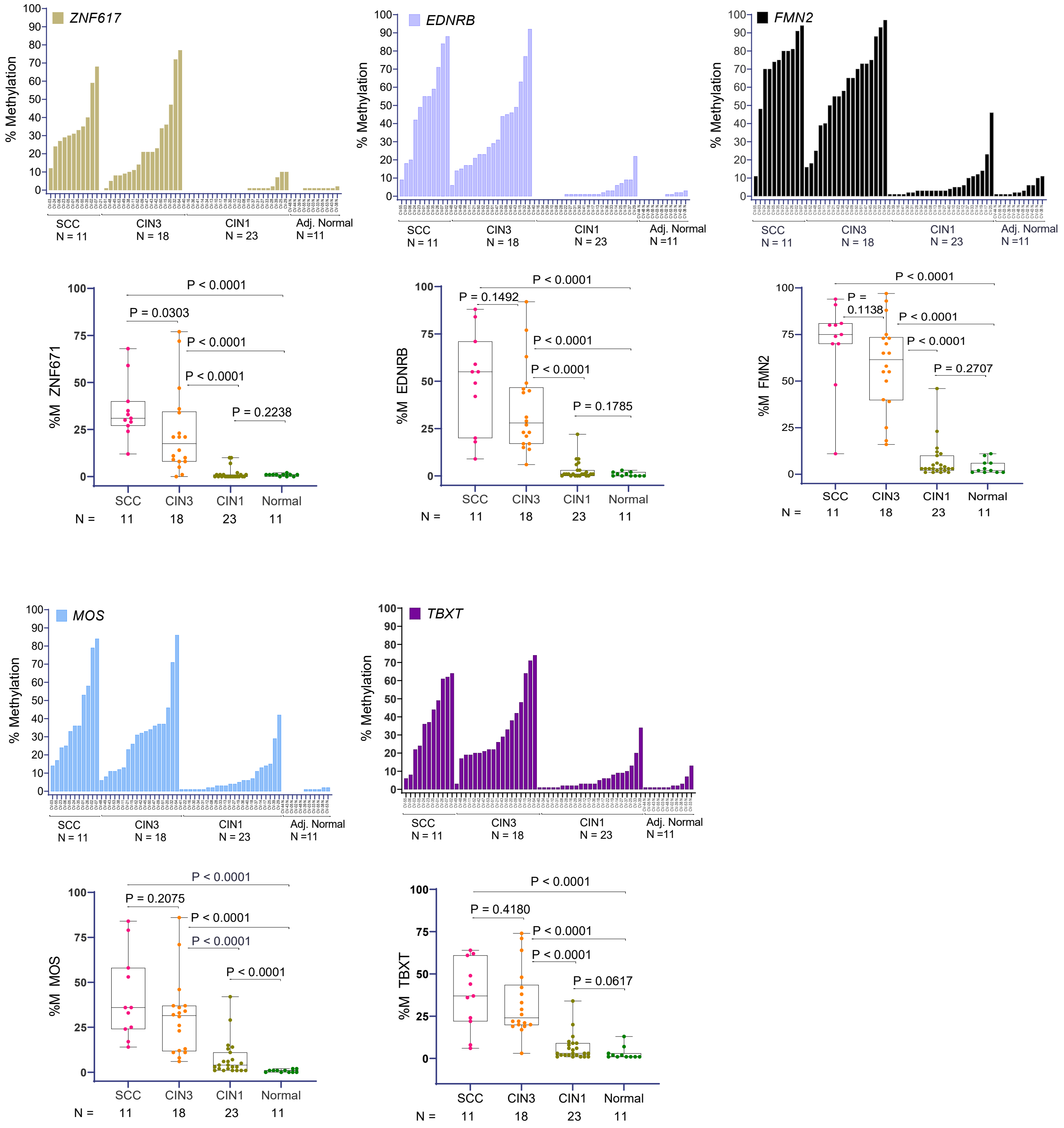

Figure S4. Contribution of individual markers of the 5-marker panel to detect cervical neoplasia. QM-MSP data of macrodissected cervical FFPE tissue (U.S. samples, N = 63) shown in main Figure 3A, was evaluated to assess the performance of each marker in progressive stages of neoplasia. Histogram plots show the magnitude of methylation (Y-axis) in each sample (X-axis). Below each histogram the corresponding box and whiskers plot indicates increasing methylation with increasing grade of disease (P < 0.0001, Mann Whitney) .

Figure S5

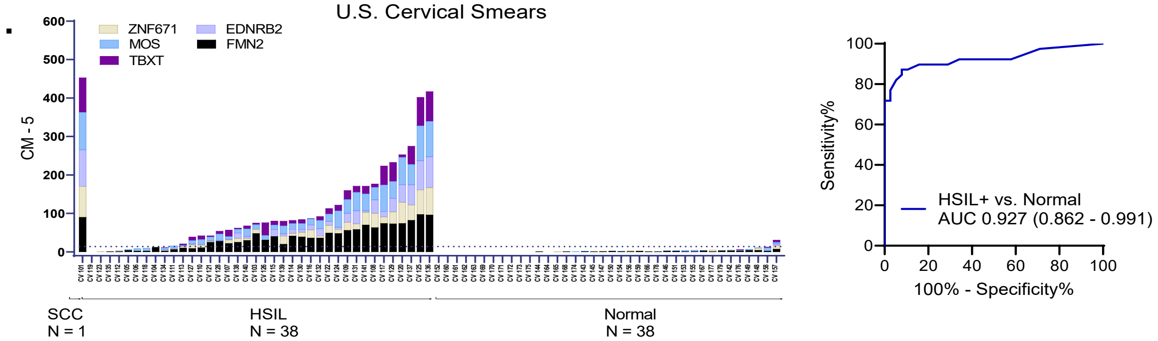

ty%	95% CI
	82.71 to 99.06

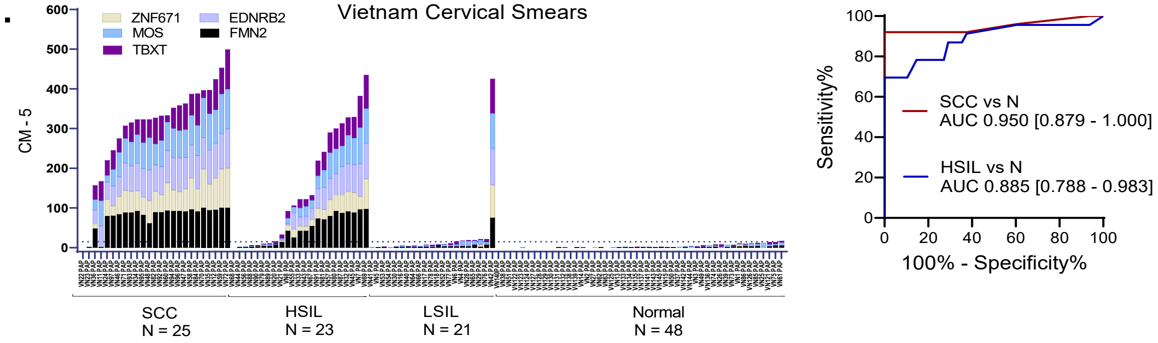

ty%	95% CI
	83.16 to 97.85
	83.16 to 97.85

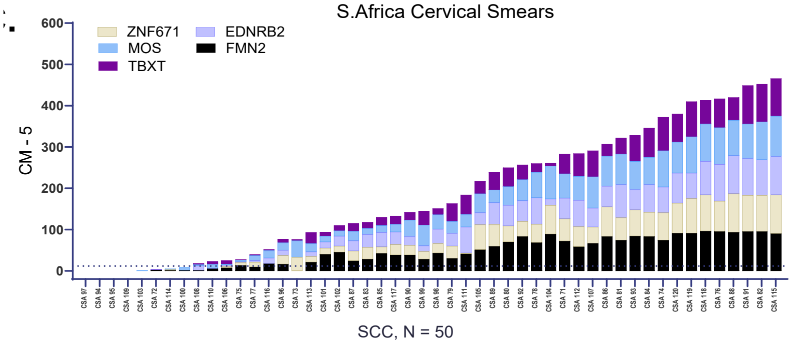

Figure S5. Detection of cervical cancer and high-grade lesions in cervical smears in each of three countries, U.S., Vietnam and S. Africa. The QM-MSP data for cervical smears from **A.** U.S. (N = 77), **B.** Vietnam (N = 117), **C.** S. Africa (N = 50) were analyzed separately by region (shown as pooled data in main Figure 4). The histogram bar height indicates the magnitude of cumulative methylation (Y-axis) in each sample (X-axis). The size of each colored segment indicates the %M for each marker. ROC analyses show high sensitivity, specificity and AUC to detect HSIL and SCC compared to normal at a threshold of the 95th percentile of CM in normal in U.S. and Vietnam (dotted line in histogram). In C., Normal cervical smear samples from S. Africa were not provided. Performance of individual markers in samples from the U.S. is shown in Figure S7.

Figure S6

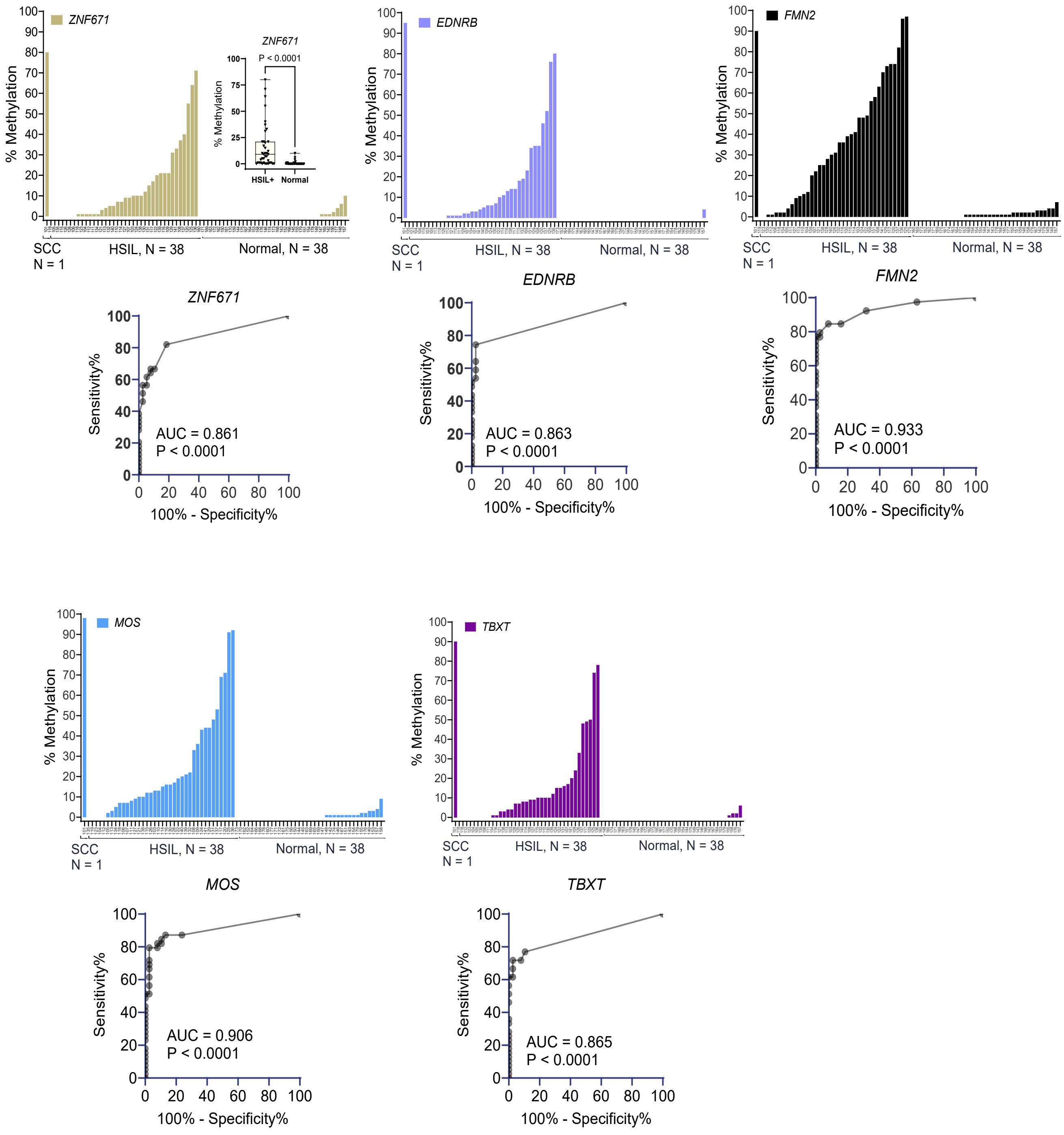

Figure S6. The 5-marker panel is highly methylated in cervical smears from patients with HSIL and SCC. Histogram analysis shows the magnitude of methylation in individual markers of the 5-marker panel obtained by QM-MSP in cervical smear samples from the U.S. (N = 77). Magnitude of methylation is shown by the bar height (%M; Y-axis) for each sample (X-axis). ROC AUC analyses shown below each histogram indicate the discriminatory ability of the marker (HSIL and SCC vs. normal), which ranged from 0.861 to 0.933, ROC P < 0.0001.

Figure S7

A.

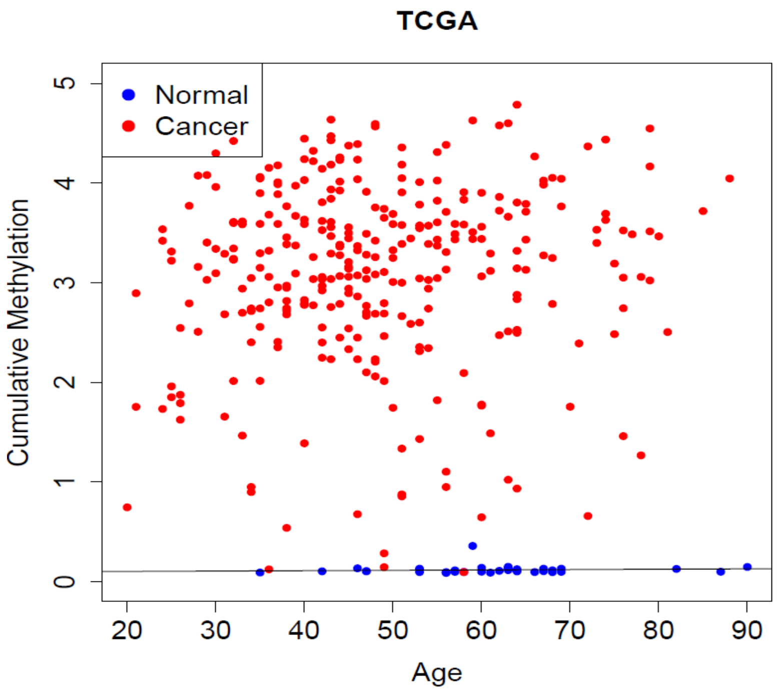

B.

Normal/Benign	Intercept	Age Coefficient	Pearson Correlation	P-value
TCGA Uterus	0.095	0.00037	0.092	0.604

Figure S7. Association between DNA methylation and age in normal/benign tissue in the TCGA UCEC database. Linear regression analysis of the effect of age on DNA methylation levels in our 5-marker panel is shown. **A**. Results are shown as plots for normal uterine tissue from TCGA. **B**. Table. Age Coefficient is the change in DNA methylation level associated with a one-year increase in age.

SUPPLEMENTAL TABLES

Table S1. Primer probe sequences for the 5-genes in the cervical cancer detection panel

PRIMER NAME	5' TO 3' DNA SEQUENCE
EDNRB_F1_Ext	TTGTTTGATGGTAGTAGAGATT
EDNRB_R1_Ext	ACTAAATTCCAACCTACTCTAA
EDNRB_FM1	GAGATTTCGAGTAAACGGTGG
EDNRB_RM1	CTTAAACTACAAACGAATCCGA
EDNRB_FUM1	GAGATTTTGAGTAAATGGTGGAG
EDNRB_RUM1	CTTAAACTACAAACAAATCCAACT
EDNRB_M1_Probe	TAACGAAACGCTACGAAAATACCAAAC
EDNRB_U1_Probe	TAACAAAACACTACAAAAATACCAAACAAT
FMN2_F2 Ext	TTGTATTATGGGGAATTAGGAT
FMN2_R2 Ext	CTTTATAACTTCCACATCCCT
FMN2_FM2	GGAAGTTGAAGAGGAGCGTAG
FMN2_RM2	CCCCAACGCATCCTCGACG
FMN2_FUM2	TGGGAAGTTGAAGAGGAGTGTA
FMN2_RUM2	AAACCCCAACACATCCTCAACA
FMN2_M2_Probe	ACCGCCGCCTTCGTACAAAACATC
FMN2_U2_Probe	ACCACCACCTTCATACAAAACATCAC
MOS_EXT_F	AGTAAGTGAATAAGTGTATTAAG
MOS_EXT_R	CCCTAAACTATTAAACCCTAC
MOS_FM1	ATTAAGAATCGATTAGTATTTCGG
MOS_RM1	TACGCGTACTAACAACCACCA
MOS_FUM1	AATTGATTAGTATTTTGGTGGAGT
MOS_RUM1	CATACACATACTAACAACCACC
MOS_M_Probe	ATTATCGTAACGCAACCTTACTACGT
MOS_U_Probe	ATCATAACACAACCTTACTACATTAAAC
TBXT_F2_Ext	GGAAGGTGGATTTTAGGTAG
TBXT_R2_Ext	CTACCCRCCTACAACTCATT
TBXT_FM2	GTCGGACGGGAGGATGAGTT
TBXT_RM2	TCCACGACGCTCAACAAATAAT
TBXT_FUM2	AGTTGGATGGGAGGATGAGTT
TBXT_RUM2	CTCCACAACACTCAACAAATAAT
TBXT M2_Probe	AAACTCTTTCCCGCGCTCTCGATA
TBXT U2_Probe	AACTCTTTCCCACACTCTCAATACC
ZNF671_EXT_F	TAGGTGGAGGTGTTGGGAAA
ZNF671_EXT_R	CTATCCTAAAACACAAAAACTAC
ZNF671_FM1	GTGTTTCGAGACGCGTTTGATG
ZNF671_RM1	AACTACCGAAAACGACAAACGTC
ZNF671_M_Probe	ATCGAAAACGCAAACACTTCCGTCC
ZNF671_FUM1	TTAGTGTTTTGAGATGTGTTTGATG
ZNF671_RUM	ACAACTACCAAAAACAACAAACATC
ZNF671_U_Probe	AATCAAAAACACAAACACTTCCATCCCT

Table S2. Descriptive statistics for 5-marker panel

Probe ID	SYMBOL	Mann Whitney p-value T/N	Ratio T/N Median	Tumor Median	Normal Median	Ratio T/N Mean
cg06971129	*EDNRB*	6.38E-23	26.7	0.594	0.022	6.90
cg25208017	*FMN2*	8.57E-22	26.6	0.675	0.025	5.79
cg15836635	*MOS*	3.97E-22	21.8	0.672	0.031	6.08
cg06073449	*TBXT*	4.09E-22	19.3	0.622	0.032	6.06
cg12074025	*ZNF671*	4.87E-21	15.8	0.682	0.043	5.22

Table S2. Descriptive statistics for 5-marker panel comparing beta methylation in cervical tumor to normal/benign tissues in TCGA database.

Table S3. TCGA-CESC Cervical marker ID, gene name, location and function

	Probe ID Cervical Cancer Discovery Set*	Probe ID QM-MSP**	Gene Name	Probe location Discovery Set***	Probe location QM-MSP	Gene Function
EDNRB	cg06971129	cg02147695	Endothelin receptor type B	Ch13:78493066; 1st Exon, CpG Island	Ch13:78493657; TSS 1500 bp, S_Shore	G-protein coupled receptor, activates the phosphatidyl-inositol-calcium signaling cascade. Aberrantly expressed and differentially methylated in cancer [1]
TBXT	cg06073449	cg11338643	T-box transcription factor T; brachyury	Ch6:166582310; TSS 200 bp, CpG Island	Ch6:166580983; Gene body, CpG Island	Embryonic nuclear DNA-binding transcription factor, apparent role in promoting tumorigenesis, progression, via activation of EMT [2]
MOS	cg15836635	cg22411207	MOS proto-oncogene, serine/threonine kinase	Ch8:57025662; 1st Exon, CpG Island	Ch8:57026301; 1st Exon, CpG Island	Protein kinase, activates MAPK signaling, aneuploidy/polyploidy in cancer cells [3]; regulates the actin cytoskeleton, essential for meiotic metaphase I and release from metaphase II [4,5]
ZNF671	cg12074025		Zinc finger protein 671	Ch19:58238850; 1st Exon, CpG Island		DNA-binding protein, transcription factor [6,7]
FMN2	cg25208017		Formin 2	Ch1:240255486; 1st Exon, CpG Island		Actin binding protein, regulates actin networks and cell polarity [8]; cyclin-dependent kinase inhibitor p21 [9]; essential for meiotic metaphase I [10]

*CpG probe location in the TCGA CESC, -UCEC database which was used for discovery of the 5-marker panel.
**Genomic location used for designing QM-MSP primers and probes. known gene functions.
*** Genome Reference Consortium Human Build 37 (GRCh37)
Abbreviations: TSS, transcriptional start site, EMT, epithelial mesenchymal transition

References for Table S3.

1. Ma X, Liu J, Wang H, Jiang Y, Wan Y, Xia Y*, et al.* Identification of crucial aberrantly methylated and differentially expressed genes related to cervical cancer using an integrated bioinformatics analysis. Biosci Rep **2020**;40

2. Chen M, Wu Y, Zhang H, Li S, Zhou J, Shen J. The Roles of Embryonic Transcription Factor BRACHYURY in Tumorigenesis and Progression. Front Oncol **2020**;10:961

3. Vitale I, Senovilla L, Jemaa M, Michaud M, Galluzzi L, Kepp O*, et al.* Multipolar mitosis of tetraploid cells: inhibition by p53 and dependency on Mos. EMBO J **2010**;29:1272-84

4. Erenpreisa J, Cragg MS. MOS, aneuploidy and the ploidy cycle of cancer cells. Oncogene **2010**;29:5447-51

5. Acevedo N, Smith GD. Oocyte-specific gene signaling and its regulation of mammalian reproductive potential. Front Biosci **2005**;10:2335-45

6. Wang Y, Chen FR, Wei CC, Sun LL, Liu CY, Yang LB*, et al.* Zinc finger protein 671 has a cancer-inhibiting function in colorectal carcinoma via the deactivation of Notch signaling. Toxicol Appl Pharmacol **2023**;458:116326

7. Zhan W, Li Y, Liu X, Zheng C, Fu Y. ZNF671 Inhibits the Proliferation and Metastasis of NSCLC via the Wnt/beta-Catenin Pathway. Cancer Manag Res **2020**;12:599-610

8. Kundu T, Siva Das S, Sewatkar LK, Kumar DS, Nagar D, Ghose A. Antagonistic Activities of Fmn2 and ADF Regulate Axonal F-Actin Patch Dynamics and the Initiation of Collateral Branching. J Neurosci **2022**;42:7355-69

9. Yamada K, Ono M, Perkins ND, Rocha S, Lamond AI. Identification and functional characterization of FMN2, a regulator of the cyclin-dependent kinase inhibitor p21. Mol Cell **2013**;49:922-33

10. Tsuiko O, Noukas M, Zilina O, Hensen K, Tapanainen JS, Magi R*, et al.* Copy number variation analysis detects novel candidate genes involved in follicular growth and oocyte maturation in a cohort of premature ovarian failure cases. Hum Reprod **2016**;31:1913-25

Table S4.

	EDNRB				ZNF671				FMN2			
Tissue diagnosis	SCC	CIN3	CIN1	N	SCC	CIN3	CIN1	N	SCC	CIN3	CIN1	N
Number of samples	11	18	23	11	11	18	23	11	11	18	23	11
**%M per sample**												
Minimum	9	6	0	0	12	0	0	0	11	16	1	1
25th percentile	20	17	0	0	27	8	0	0	70	40	2	1
Median	55	28	1	0	31	18	0	1	75	62	3	2
75th percentile	71	47	3	2	40	35	1	1	81	74	10	6
Maximum	88	92	22	3	68	77	10	2	94	97	46	11
P-value (SCC vs. N)	< 0.0001				< 0.0001				< 0.0001			
P-value (CIN3 vs. N)	< 0.0001				< 0.0001				< 0.0001			
P-value (SCC vs. CIN3)	0.1492				0.0303				0.1138			

SCC, squamous cell carcinoma; CIN3, cervical intraepithelial neoplasia grade3, CIN2,cervical intraepithelial neoplasia grade2, N, adjacent normal tissue; P value for Mann Whitney test.

	MOS				TBXT			
Tissue diagnosis	SCC	CIN3	CIN1	N	SCC	CIN3	CIN1	N
Number of samples	11	18	23	11	11	18	23	11
**%M per sample**								
Minimum	14	6	1	0	6	3	1	1
25th percentile	24	12	1	0	22	20	2	1
Median	36	32	4	1	37	24	3	1
75th percentile	58	37	11	1	61	44	9	3
Maximum	84	86	42	2	64	74	34	13
P-value (SCC vs. N)	< 0.0001				< 0.0001			
P-value (CIN3 vs. N)	< 0.0001				< 0.0001			
P-value (SCC vs. CIN3)	0.2075				0.4180			

SCC, squamous cell carcinoma; CIN3, cervical intraepithelial neoplasia grade3, CIN2,cervical intraepithelial neoplasia grade2, N, adjacent normal tissue; P value for Mann Whitney test.

Table S4. Descriptive statistics for methylation of individual CM-5 markers in tissue. FFPE tissue sections from the U.S. (N = 63) were macrodissected and methylation was quantified using QM-MSP for *EDNRB, ZNF671, FMN2, MOS* and *TBXT.* Data was analyzed using Mann Whitney statistic for the indicated comparisons. Figure S4 accompanies this table.

Table S5.

SAMPLES USED IN THIS STUDY			
**Set 1 Tissue**	United States - Macrodissected	Vietnam	South Africa
SCC	11	31	23
CIN2/3	18	31	23
LSIL (CIN1)	23	28	0
Benign	0	30	23
Benign adjacent to tumor	11	0	0
Total	63	120	69
**Set 2 Cervical Smear**	United States	Vietnam	South Africa
SCC	1	25	50
HSIL	38	23	0
LSIL	0	21	0
Normal	38	48	0
Total	77	117	50
**Paired subsets**	PAIRED (United States)	PAIRED (Vietnam)	PAIRED (South African)
SCC smear + tissue	0	25	0
HSIL smear + tissue	0	23	0
LSIL smear + tissue	0	21	0
Benign smear + tissue	0	23	0
Total	0	92	0

S5 Detailed description of samples used in this study. Quantitative Multiplex-Methylation Specific PCR (QM-MSP) was performed on archival formalin fixed paraffin embedded (FFPE)-tissue and cervical smear samples. Paired smear/tissue samples were available from Vietnam, a subset the Vietnam samples shown in Set 1 and Set 2. This information supports Figure 1 and 3 and sample demographics provided in S6 and S7 tables. SCC- Squamous cell carcinoma; CIN2/3-Cervical intraepithelial neoplasia 2/3; CIN1**-** Cervical intraepithelial neoplasia 1; HSIL- High grade intraepithelial lesion, LSIL-low grade intraepithelial lesion; CIN2- Cervical intraepithelial neoplasia 2.

Table S6. United States Patient Characteristics

S5 Table. United States Patient Characteristics						
**Set 1 Formalin Fixed Paraffin Embedded Tissue, Macrodissected Tissues**						
**CV ID**	**DX**	**Age**	**HPV**	**Stage of cancer**	**Follow-up (momth)**	**Outcome**
CV 01	SCC	42	Not available	1A1	87	alive
CV 02	SCC	38	HPV16	1B1	33	alive
CV 03	SCC	34	Not available	1A1	65	alive
CV 05	SCC	69	Detected (not typed)	1B	84	alive
CV 06	SCC	63	HPV16	IV	66	alive
CV 07	SCC	76	HPV16	2B	58	alive
CV 23	SCC	70	HPV16, 18	Not available	Not available	Not available
CV 24	SCC	47	HPV16	2B	58	alive
CV 26	SCC	50	HPV16	2B	60	alive
CV 35	SCC	52	HPV16	IV	58	alive
CV 55	SCC	65	HPV16	1B	49	alive
CV 09	CIN 3	38	HPV16	-	58	alive
CV 11	CIN 3	43	HPV16	-	61	alive
CV 20	CIN 3	51	Not available	-	48	alive
CV 21	CIN 3	28	Not available	-	24	alive
CV 32	CIN 3	27	Not available	-	58	alive
CV 38	CIN 3	29	Not detected	-	47	alive
CV 42	CIN 3	29	HPV16 and non 16/18	-	54	alive
CV 43	CIN 3	31	HPV16	-	33	alive
CV 44	CIN 3	30	HPV16	-	55	alive
CV 45	CIN 3	45	Detected (not 16 or 18)	-	55	alive
CV 47	CIN 3	42	Detected (not 16 or 18)	-	19	dead
CV 48	CIN 3	31	HPV16	-	48	alive
CV 49	CIN 3	25	Detected (not 16 or 18)	-	43	alive
CV 50	CIN 3	58	HPV16	-	3	alive
CV 51	CIN 3	31	HPV18	-	20	alive
CV 52	CIN 3	24	HPV16	-	24	alive
CV 53	CIN 3	29	HPV16	-	54	alive
CV 54	CIN 3	27	Detected (not typed)	-	52	alive
CV 08	CIN 1	25	Detected (not 16 or 18)	-	47	alive
CV 10	CIN 1	44	Detected (not 16 or 18)	-	60	alive
CV 12	CIN 1	26	Detected (not 16 or 18)	-	60	alive
CV 13	CIN 1	29	Detected (not 16 or 18)	-	60	alive
CV 14	CIN 1	26	Not available	-	48	alive
CV 15	CIN 1	36	HPV18	-	41	alive
CV 16	CIN 1	31	Detected (not 16 or 18)	-	60	alive
CV 17	CIN 1	32	Detected HPV16, 18, non 16/18	-	59	alive
CV 18	CIN 1	35	Detected (not 16 or 18)	-	59	alive
CV 19	CIN 1	44	Detected (not 16 or 18)	-	59	alive
CV 22	CIN 1	38	Detected (not 16 or 18)	-	59	alive
CV 25	CIN 1	64	Detected (not 16 or 18)	-	57	alive
CV 27	CIN 1	36	HPV18	-	59	alive
CV 28	CIN 1	27	Detected (not 16 or 18)	-	58	alive
CV 29	CIN 1	37	Detected (not 16 or 18)	-	44	alive
CV 30	CIN 1	31	Detected (not 16 or 18)	-	57	alive
CV 31	CIN 1	25	Not available	-	43	alive
CV 33	CIN 1	39	Detected (not 16 or 18)	-	Not available	Not available
CV 34	CIN 1	69	HPV16 and non 16/18	-	59	alive
CV 36	CIN 1	39	Detected (not 16 or 18)	-	58	alive
CV 37	CIN 1	44	Detected (not 16 or 18)	-	55	alive
CV 39	CIN 1	44	Detected (not typed)	-	57	alive
CV 40	CIN 1	29	Detected (not 16 or 18)	-	57	alive
CV 41	CIN 1	27	Detected (not typed)	-	52	alive

Table S6. United States Patient Characteristics (cont.)

Set 2 Cervical Smears						
CV ID	DX	Age	HPV	Stage of cancer	Follow-up (momth)	Outcome
CV 101	SCC	59	Detected (not typed)	2B	84	alive
CV 103	HSIL	44	Not available	-	27	alive
CV 104	HSIL	39	Not detected	-	57	alive
CV 105	HSIL	26	Detected (not typed)	-	103	alive
CV 106	HSIL	42	Not available	-	104	alive
CV 107	HSIL	29	Equivocal	-	9	alive
CV 108	HSIL	36	HPV18 (not 16)	-	104	alive
CV 109	HSIL	60	Not available	-	15	dead
CV 110	HSIL	32	Detected (not typed)	-	25	alive
CV 111	HSIL	32	Not available	-	89	alive
CV 112	HSIL	28	Detected (not typed)	-	101	alive
CV 113	HSIL	37	Detected (not typed)	-	103	alive
CV 114	HSIL	38	Detected (not typed)	-	52	alive
CV 115	HSIL	22	Not available	-	104	alive
CV 116	HSIL	31	Detected (not typed)	-	100	alive
CV 117	HSIL	40	Not available	-	17	dead
CV 118	HSIL	53	Not available	-	103	alive
CV 119	HSIL	24	Not available	-	99	alive
CV 120	HSIL	35	Detected (not typed)	-	Not available	Not available
CV 121	HSIL	33	Detected (not typed)	-	100	alive
CV 122	HSIL	24	Not available	-	88	alive
CV 123	HSIL	29	Detected (not typed)	-	96	alive
CV 124	HSIL	26	Detected (not 16/18)	-	103	alive
CV 125	HSIL	56	Detected (not 16/18)	-	103	alive
CV 126	HSIL	24	Detected (not typed)	-	93	alive
CV 127	HSIL	38	Detected (not typed)	-	10	alive
CV 128	HSIL	37	Not detected	-	100	alive
CV 129	HSIL	26	Detected (not typed)	-	99	alive
CV 130	HSIL	34	Detected (not typed)	-	98	alive
CV 131	HSIL	50	Detected (not typed)	-	100	alive
CV 132	HSIL	40	Detected (not typed)	-	104	alive
CV 134	HSIL	59	Detected (not typed)	-	103	alive
CV 135	HSIL	31	detected (not typed)	-	95	alive
CV 136	HSIL	42	Detected (not typed)	-	79	alive
CV 137	HSIL	33	Detected (not typed)	-	100	alive
CV 138	HSIL	23	Not available	-	90	alive
CV 139	HSIL	26	Not available	-	Not available	Not available
CV 140	HSIL	40	Not available	-	24	alive
CV 141	HSIL	42	Detected (not typed)	-	87	alive
CV 142	Normal	43	Not detected	-	95	alive
CV 143	Normal	37	Not detected	-	101	alive
CV 144	Normal	57	Not detected	-	102	alive
CV 145	Normal	37	Not detected	-	94	alive
CV 146	Normal	39	Not detected	-	102	alive
CV 147	Normal	38	Not detected	-	84	alive
CV 148	Normal	57	Detected (not 16/18)	-	88	alive
CV 149	Normal	33	Not detected	-	16	alive
CV 150	Normal	43	Not detected	-	88	alive
CV 151	Normal	50	Not detected	-	102	alive
CV 152	Normal	30	Not detected	-	8	alive
CV 153	Normal	34	Not detected	-	102	alive
CV 154	Normal	49	Not detected	-	87	alive
CV 155	Normal	39	Not detected	-	80	alive
CV 156	Normal	37	Not detected	-	88	alive
CV 157	Normal	51	Not detected	-	101	alive
CV 158	Normal	33	Not detected	-	65	alive
CV 159	Normal	29	Not detected	-	96	alive
CV 160	Normal	28	Not available	-	Not available	Not available
CV 161	Normal	26	Not detected	-	94	alive
CV 162	Normal	22	Not available	-	76	alive
CV 163	Normal	20	Not available	-	102	alive
CV 164	Normal	21	Not available	-	78	alive
CV 165	Normal	21	Not available	-	70	alive
CV 166	Normal	22	Not available	-	22	alive
CV 167	Normal	23	Not available	-	8	alive
CV 168	Normal	29	Not available	-	Not available	Not available
CV 169	Normal	22	Detected (not typed)	-	102	alive
CV 170	Normal	21	Not available	-	51	alive
CV 171	Normal	23	Not available	-	102	alive
CV 172	Normal	26	Not detected	-	102	alive
CV 173	Normal	24	Not available	-	102	alive
CV 174	Normal	28	Not detected	-	58	alive
CV 175	Normal	28	Not available	-	91	alive
CV 176	Normal	35	Not detected	-	89	alive
CV 177	Normal	50	Not detected	-	33	alive
CV 178	Normal	35	Not detected	-	101	alive
CV 179	Normal	36	Not detected	-	82	alive

Table S7. Vietnam Sample Demographic Data

S6 Table. Vietnam Patient Characteristics

Diagnosis (Histology)	Tissue ID	Cervical smear ID	Age	Formalin Fixed Paraffin Embedded Tissue (FFPE) Diagnosis	Cervical Smear Cytology Diagnosis	Histology details	Grade	Stage	Treatment	Chemotherpay	Radiation
SCC	VN11 T	VN11 PAP	44	Carcinoma	Carcinoma	Nonkeratinizing squamous cell, invasive	II	II	No surgery	Yes	Yes
SCC	VN12 T	VN12 PAP	42	Carcinoma	Carcinoma	Nonkeratinizing squamous cell, invasive	II	Ib	Hysterectomy	No	No
SCC	VN22 T	VN22 PAP	56	Carcinoma	Carcinoma	Nonkeratinizing squamous cell, invasive	II	III	No surgery	No	No
SCC	VN23 T	VN23 PAP	42	Carcinoma	Carcinoma	Nonkeratinizing squamous cell, invasive	II	IV	No surgery	Yes	Yes
SCC	VN24 T	VN24 PAP	53	Carcinoma	Carcinoma	Nonkeratinizing squamous cell, invasive	II	IV	Hysterectomy	Yes	Yes
SCC	VN34 T	VN34 PAP	40	Carcinoma	Carcinoma	Keratinizing squamous cell, invasive	II	II	No surgery	Yes	Yes
SCC	VN35 T	VN35 PAP	60	Carcinoma	Carcinoma	Nonkeratinizing squamous cell, invasive	II	II	No surgery	Yes	Yes
SCC	VN36 T	VN36 PAP	40	Carcinoma	Carcinoma	Nonkeratinizing squamous cell, invasive	II	II	Hysterectomy	No	No
SCC	VN46 T	VN46 PAP	57	Carcinoma	Carcinoma	Nonkeratinizing squamous cell, invasive	II	Not available	Not available	Not available	Not available
SCC	VN47 T	VN47 PAP	60	Carcinoma	Carcinoma	Keratinizing squamous cell, invasive	II	II	No surgery	Yes	Yes
SCC	VN48 T	VN48 PAP	57	Carcinoma	Carcinoma	Adenosquamous cell carcinoma	-	III	No surgery	No	Yes
SCC	VN58 T	VN58 PAP	54	Carcinoma	Carcinoma	Nonkeratinizing squamous cell, invasive	II	II	No surgery	Yes	Yes
SCC	VN59 T	VN59 PAP	57	Carcinoma	Carcinoma	Nonkeratinizing squamous cell, invasive	II	IIIa	No surgery	Yes	Yes
SCC	VN60 T	VN60 PAP	61	Carcinoma	Carcinoma	Nonkeratinizing squamous cell, invasive	II	IIa	No surgery	Yes	Yes
SCC	VN70 T	VN70 PAP	57	Carcinoma	Carcinoma	Squamous cell carcinoma, invasive	II	IV	No surgery	Yes	Yes
SCC	VN71 T	VN71 PAP	52	Carcinoma	Carcinoma	Invasive squamous cell carcinoma, invasive	II	IV	Hysterectomy	Yes	Yes
SCC	VN72 T	VN72 PAP	54	Carcinoma	Carcinoma	Squamous cell carcinoma	II	Not available	Not available	Not available	Not available
SCC	VN82 T	VN82 PAP	67	Carcinoma	Carcinoma	Squamous cell carcinoma, invasive	II	II	Hysterectomy	Yes	Yes
SCC	VN84 T	VN84 PAP	48	Carcinoma	Carcinoma	Nonkeratinizing squamous cell, invasive	II	IIb-III	No surgery	Yes	Yes
SCC	VN92 T	VN92 PAP	68	Carcinoma	Carcinoma	Nonkeratinizing squamous cell, invasive	II	II	No surgery	Yes	Yes
SCC	VN93 T	VN93 PAP	63	Carcinoma	Carcinoma	Nonkeratinizing Squamous cell	-	II	No surgery	Yes	Yes
SCC	VN94 T	VN94 PAP	42	Carcinoma	Carcinoma	Keratinizing squamous cell, invasive	II	II	Hysterectomy	No	Yes
SCC	VN95 T	VN95 PAP	46	Carcinoma	Carcinoma	Keratinizing squamous cell, invasive	II	IV	Hysterectomy	Yes	Yes
SCC	VN96 T	VN96 PAP	64	Carcinoma	Carcinoma	Squamous cell carcinoma	II	III	No surgery	Yes	Yes
SCC	VN97 T	VN97 PAP	68	Carcinoma	Carcinoma	Nonkeratinizing squamous cell, invasive	III	III	No surgery	Yes	Yes
SCC	VN83 T	-	71	Carcinoma	-	Squamous cell carcinoma	II	II	No surgery	No	No
SCC	VN119 T	-	62	Carcinoma	-	Squamous cell carcinoma	-	Not available	Not available	Not available	Not available
SCC	VN10 T	-	58	Carcinoma	-	Nonkeratinizing squamous cell, invasive	II	IIIb	No surgery	Yes	Yes
SCC	VN118 T	-	74	Carcinoma	-	Nonkeratinizing squamous cell, invasive	II	Not available	Not available	Not available	Not available
SCC	VN150 T	-	45	Carcinoma	-	Nonkeratinizing squamous cell, invasive	III	I	Hysterectomy	No	No
SCC	VN117 T	-	43	Carcinoma	-	Nonkeratinizing squamous cell, invasive	II	Not available	Not available	Not available	Not available

Diagnosis (Histology)	Tissue ID	Cervical smear ID	Age	FFPE Tissue Diagnosis	Cervical Smear Cytology Diagnosis	Histology details	Grade	Stage	Treatment	Chemotherpay	Radiation
HSIL	VN20 T	VN20 PAP	40	HSIL	HSIL	CIN 3	-	-	Leep and follow-up	-	-
HSIL	VN21 T	VN21 PAP	35	HSIL	HSIL	CIN 3	-	-	Not available	-	-
HSIL	VN32 T	VN32 PAP	38	HSIL	HSIL	CIN 3	-	II	No Surgery	Yes	Yes
HSIL	VN33 T	VN33 PAP	40	HSIL	HSIL	CIN 3	-	-	Leep and follow-up	-	-
HSIL	VN43 T	VN43 PAP	39	HSIL	HSIL	CIN 3	-	-	Not available	-	-
HSIL	VN44 T	VN44 PAP	24	HSIL	HSIL	CIN 3	-	-	Leep and follow-up	-	-
HSIL	VN45 T	VN45 PAP	46	HSIL	HSIL	CIN 3	-	-	Not available	-	-
HSIL	VN55 T	VN55 PAP	37	HSIL	HSIL	CIN 3	-	-	Leep and follow-up	-	-
HSIL	VN56 T	VN56 PAP	43	HSIL	HSIL	CIN 3	-	I	Hysterectomy	Yes	Yes
HSIL	VN57 T	VN57 PAP	34	HSIL	HSIL	CIN 3	-	-	Leep and follow-up	-	-
HSIL	VN67 T	VN67 PAP	44	HSIL	HSIL	CIN 3	-	-	Leep and follow-up	-	-
HSIL	VN68 T	VN68 PAP	65	HSIL	HSIL	CIN 3	-	-	Hysterectomy, follow-up	-	-
HSIL	VN69 T	VN69 PAP	52	HSIL	HSIL	CIN 3	-	II	No Surgery	Yes	Yes
HSIL	VN7 T	VN7 PAP	64	HSIL	HSIL	HSIL	-	I	Hysterectomy, follow-up	No	No
HSIL	VN79 T	VN79 PAP	54	HSIL	HSIL	CIN 2	-	-	Leep and follow-up	-	-
HSIL	VN8 T	VN8 PAP	50	HSIL	HSIL	HSIL	-	I	Leep and follow-up	-	-
HSIL	VN80 T	VN80 PAP	38	HSIL	HSIL	CIN 3	-	-	Leep and follow-up	-	-
HSIL	VN81 T	VN81 PAP	43	HSIL	HSIL	CIN 3	-	-	Leep and follow-up	-	-
HSIL	VN87 T	VN87 PAP	44	HSIL	HSIL	CIN 3	-	II	Hysterectomy, follow-up	Yes	Yes
HSIL	VN88 T	VN88 PAP	54	HSIL	HSIL	CIN 3	-	-	Leep and follow-up	-	-
HSIL	VN89 T	VN89 PAP	41	HSIL	HSIL	CIN 2	-	-	Not available	-	-
HSIL	VN90 T	VN90 PAP	38	HSIL	HSIL	CIN 2	-	-	Not available	-	-
HSIL	VN91 T	VN91 PAP	43	HSIL	HSIL	CIN 3	-	-	Leep and follow-up	-	-
HSIL	VN115 T	-	49	HSIL	-	CIN 3	-	-	Hysterectomy, follow-up	No	No
HSIL	VN31 T	-	49	HSIL	-	CIN 3	-	-	Hysterectomy, follow-up	-	-
HSIL	VN9 T	-	27	HSIL	-	HSIL	-	-	Leep and follow-up	-	-
HSIL	VN19 T	-	33	HSIL	-	CIN 3	-	-	Leep and follow-up	-	-
HSIL	VN114 T	-	45	HSIL	-	CIN 2	-	-	Not available	-	-
HSIL	VN116 T	-	24	HSIL	-	CIN 2	-	-	Not available	-	-
HSIL	VN113 T	-	41	HSIL	-	CIN 2	-	-	Not available	-	-
HSIL	VN112 T	-	43	HSIL	-	CIN 3	-	-	Leep and follow-up	-	-

Table S7. Vietnam Sample Demographic Data (cont.)

Diagnosis (Histology)	Tissue ID	Cervical smear ID	Age	FFPE Tissue Diagnosis	Cervical Smear Cytology Diagnosis	Histology details	Grade	Stage	Treatment	Chemotherpay	Radiation
LSIL	VN16 T	VN16 PAP	43	LSIL	LSIL	LSIL	-	-	Leep and follow-up	-	-
LSIL	VN17 T	VN17 PAP	39	LSIL	LSIL	LSIL	-	-	Leep and follow-up	-	-
LSIL	VN18 T	VN18 PAP	38	LSIL	LSIL	LSIL	-	-	Leep and follow-up	-	-
LSIL	VN28 T	VN28 PAP	49	LSIL	LSIL	LSIL	-	-	Leep and follow-up	-	-
LSIL	VN29 T	VN29 PAP	53	LSIL	LSIL	LSIL	-	-	Leep and follow-up	-	-
LSIL	VN30 T	VN30 PAP	58	LSIL	LSIL	LSIL	-	-	Leep and follow-up	-	-
LSIL	VN4 T	VN4 PAP	32	LSIL	LSIL	LSIL	-	-	Leep and follow-up	-	-
LSIL	VN40 T	VN40 PAP	44	LSIL	LSIL	LSIL	-	-	Leep and follow-up	-	-
LSIL	VN41 T	VN41 PAP	45	LSIL	LSIL	LSIL	-	-	Leep and follow-up	-	-
LSIL	VN42 T	VN42 PAP	50	LSIL	LSIL	LSIL	-	-	Leep and follow-up	-	-
LSIL	VN5 T	VN5 PAP	43	LSIL	LSIL	LSIL	-	-	Leep and follow-up	-	-
LSIL	VN52 T	VN52 PAP	33	LSIL	LSIL	LSIL	-	-	Leep and follow-up	-	-
LSIL	VN53 T	VN53 PAP	36	LSIL	LSIL	LSIL	-	-	Leep and follow-up	-	-
LSIL	VN54 T	VN54 PAP	42	LSIL	LSIL	LSIL	-	-	Leep and follow-up	-	-
LSIL	VN6 T	VN6 PAP	49	LSIL	LSIL	LSIL	-	-	Leep and follow-up	-	-
LSIL	VN64 T	VN64 PAP	36	LSIL	LSIL	LSIL	-	-	Leep and follow-up	-	-
LSIL	VN65 T	VN65 PAP	41	LSIL	LSIL	LSIL	-	-	Leep and follow-up	-	-
LSIL	VN66 T	VN66 PAP	34	LSIL	LSIL	LSIL	-	-	Leep and follow-up	-	-
LSIL	VN76 T	VN76 PAP	31	LSIL	LSIL	LSIL	-	-	Leep and follow-up	-	-
LSIL	VN77 T	VN77 PAP	30	LSIL	LSIL	LSIL	-	-	Leep and follow-up	-	-
LSIL	VN78 T	VN78 PAP	45	LSIL	LSIL	LSIL	-	-	Leep and follow-up	-	-
LSIL	VN108 T	-	46	LSIL	-	LSIL	-	-	Leep and follow-up	-	-
LSIL	VN107 T	-	39	LSIL	-	LSIL	-	-	Leep and follow-up	-	-
LSIL	VN106 T	-	49	LSIL	-	LSIL	-	-	Leep and follow-up	-	-
LSIL	VN105 T	-	32	LSIL	-	LSIL	-	-	Leep and follow-up	-	-
LSIL	VN109 T	-	28	LSIL	-	LSIL	-	-	Leep and follow-up	-	-
LSIL	VN111 T	-	31	LSIL	-	LSIL	-	-	Leep and follow-up	-	-
LSIL	VN110 T	-	29	LSIL	-	LSIL	-	-	Leep and follow-up	-	-

Diagnosis (Histology)	Tissue ID	Cervical smear ID	Age	FFPE Tissue Diagnosis	Cervical Smear Cytology Diagnosis	Histology details	Grade	Stage	Treatment	Chemotherpay	Radiation
Benign	VN1 T	VN1 PAP	43	Benign	Normal	Benign					
Benign	VN13 T	VN13 PAP	54	Benign	Normal	Benign					
Benign	VN14 T	VN14 PAP	41	Benign	Normal	Benign					
Benign	VN15 T	VN15 PAP	33	Benign	Normal	Benign					
Benign	VN2 T	VN2 PAP	51	Benign	Normal	Benign					
Benign	VN25 T	VN25 PAP	40	Benign	Normal	Benign					
Benign	VN26 T	VN26 PAP	25	Benign	Normal	Benign					
Benign	VN27 T	VN27 PAP	35	Benign	Normal	Benign					
Benign	VN3 T	VN3 PAP	49	Benign	Normal	Benign					
Benign	VN37 T	VN37 PAP	48	Benign	Normal	Benign					
Benign	VN38 T	VN38 PAP	30	Benign	Normal	Benign					
Benign	VN39 T	VN39 PAP	22	Benign	Normal	Benign					
Benign	VN49 T	VN49 PAP	43	Benign	Normal	Benign					
Benign	VN50 T	VN50 PAP	44	Benign	Normal	Benign					
Benign	VN51 T	VN51 PAP	35	Benign	Normal	Benign					
Benign	VN61 T	VN61 PAP	46	Benign	Normal	Benign					
Benign	VN62 T	VN62 PAP	39	Benign	Normal	Benign					
Benign	VN63 T	VN63 PAP	47	Benign	Normal	Benign					
Benign	VN73 T	VN73 PAP	34	Benign	Normal	Benign					
Benign	VN74 T	VN74 PAP	39	Benign	Normal	Benign					
Benign	VN75 T	VN75 PAP	52	Benign	Normal	Benign					
Benign	VN85 T	VN85 PAP	40	Benign	Normal	Benign					
Benign	VN86 T	VN86 PAP	38	Benign	Normal	Benign					
Benign	VN101 T	-	39	Benign	-	Benign					
Benign	VN102 T	-	38	Benign	-	Benign					
Benign	VN104 T	-	33	Benign	-	Benign					
Benign	VN98 T	-	40	Benign	-	Benign					
Benign	VN103 T	-	51	Benign	-	Benign					
Benign	VN99 T	-	52	Benign	-	Benign					
Benign	VN100 T	-	35	Benign	-	Benign					

Set 3 Diagnosis Histology Central Review	Tissue ID	Cervical smear ID	Age	FFPE Tissue Diagnosis	Cervical Smear Cytology Diagnosis	Histology details	Grade	Stage	Treatment	Chemotherpay	Radiation
CIN1	VN151	-	42	CIN 2	-	-	-	-	Leep and Follow-up	-	-
CIN1	VN152	-	53	CIN 2	-	-	-	-	Leep and Follow-up	-	-
CIN1	VN154	-	43	CIN 2	-	-	-	-	Leep and Follow-up	-	-
CIN1	VN155	-	40	CIN 2	-	-	-	-	Leep and Follow-up	-	-
CIN1	VN159	-	31	CIN 2	-	-	-	-	Leep and Follow-up	-	-
CIN1	VN163	-	45	CIN 2	-	-	-	-	Leep and Follow-up	-	-
CIN1	VN165	-	31	CIN 2	-	-	-	-	Leep and Follow-up	-	-
CIN1	VN166	-	31	CIN 2	-	-	-	-	Leep and Follow-up	-	-
CIN1	VN169	-	29	CIN 2	-	-	-	-	Leep and Follow-up	-	-
CIN2	VN156	-	46	CIN 2	-	-	-	-	Leep and Follow-up	-	-
CIN2	VN158	-	30	CIN 2	-	-	-	-	Hysterectomy	-	-
CIN2	VN160	-	48	CIN 2	-	-	-	-	Hysterectomy, follow-up	-	-
CIN2	VN161	-	39	CIN 2	-	-	-	-	Leep and Follow-up	-	-
CIN2	VN167	-	32	CIN 2	-	-	-	-	Leep and Follow-up	-	-
CIN2	VN168	-	46	CIN 2	-	-	-	-	Leep and Follow-up	-	-
Benign	VN153	-	48	CIN 2	-	-	-	-	Leep and Follow-up	-	-
Benign	VN157	-	22	CIN 2	-	-	-	-	Leep and Follow-up	-	-
Benign	VN164	-	44	CIN 2	-	-	-	-	Leep and Follow-up	-	-

Table S8. HPV status of patients in the GSE68339 dataset

GSE68339 Dataset	Sample (ID)	HPV Status
GSM1675986	Tumor biopsy (P-053)	Positive
GSM1676140	Tumor biopsy (P-226)	Positive
GSM1675991	Tumor biopsy (P-067)	Positive
GSM1676116	Tumor biopsy (P-374)	Positive
GSM1676144	Tumor biopsy (P-255)	Positive
GSM1676118	Tumor biopsy (P-384)	Positive
GSM1676123	Tumor biopsy (P-392)	Positive
GSM1676103	Tumor biopsy (P-346)	Positive
GSM1675982	Tumor biopsy (P-024)	Positive
GSM1676084	Tumor biopsy (P-289)	Positive
GSM1675984	Tumor biopsy (P-032)	Positive
GSM1676156	Tumor biopsy (P-300)	Positive
GSM1676150	Tumor biopsy (P-269)	Positive
GSM1676005	Tumor biopsy (P-115)	Positive
GSM1676236	Tumor biopsy (P-525)	Positive
GSM1676178	Tumor biopsy (P-413)	Positive
GSM1676092	Tumor biopsy (P-308)	Positive
GSM1676234	Tumor biopsy (P-520)	Positive
GSM1676224	Tumor biopsy (P-505)	Positive
GSM1676193	Tumor biopsy (P-443)	Positive
GSM1676036	Tumor biopsy (P-192)	Positive
GSM1676049	Tumor biopsy (P-213)	Positive
GSM1676041	Tumor biopsy (P-197)	Positive
GSM1676244	Tumor biopsy (P-536)	Positive
GSM1676180	Tumor biopsy (P-417)	Positive
GSM1676161	Tumor biopsy (P-353)	Positive
GSM1676022	Tumor biopsy (P-151)	Positive
GSM1676003	Tumor biopsy (P-098)	Positive
GSM1676152	Tumor biopsy (P-280)	Positive
GSM1676060	Tumor biopsy (P-236)	Positive
GSM1676213	Tumor biopsy (P-483)	Positive
GSM1676050	Tumor biopsy (P-214)	Positive
GSM1676228	Tumor biopsy (P-510)	Positive
GSM1676008	Tumor biopsy (P-124)	Positive
GSM1676119	Tumor biopsy (P-387)	Positive
GSM1676143	Tumor biopsy (P-254)	Positive
GSM1676130	Tumor biopsy (P-060)	Positive
GSM1676131	Tumor biopsy (P-081)	Positive
GSM1676163	Tumor biopsy (P-358)	Positive
GSM1676069	Tumor biopsy (P-258)	Positive

GSM1676191	Tumor biopsy (P-438)	Positive
GSM1676121	Tumor biopsy (P-390)	Positive
GSM1676006	Tumor biopsy (P-120)	Positive
GSM1676067	Tumor biopsy (P-252)	Positive
GSM1676158	Tumor biopsy (P-335)	Positive
GSM1676097	Tumor biopsy (P-326)	Positive
GSM1676056	Tumor biopsy (P-228)	Positive
GSM1676217	Tumor biopsy (P-488)	Positive
GSM1676039	Tumor biopsy (P-195)	Positive
GSM1676026	Tumor biopsy (P-172)	Positive
GSM1676137	Tumor biopsy (P-207)	Positive
GSM1676048	Tumor biopsy (P-210)	Positive
GSM1676086	Tumor biopsy (P-291)	Positive
GSM1676126	Tumor biopsy (P-003)	Positive
GSM1675990	Tumor biopsy (P-063)	Positive
GSM1676201	Tumor biopsy (P-464)	Positive
GSM1676229	Tumor biopsy (P-512)	Positive
GSM1676090	Tumor biopsy (P-302)	Positive
GSM1676245	Tumor biopsy (P-537)	Positive
GSM1676206	Tumor biopsy (P-471)	Positive
GSM1676155	Tumor biopsy (P-298)	Positive
GSM1676104	Tumor biopsy (P-347)	Positive
GSM1676239	Tumor biopsy (P-529)	Positive
GSM1676053	Tumor biopsy (P-221)	Positive
GSM1676018	Tumor biopsy (P-144)	Positive
GSM1676019	Tumor biopsy (P-146)	Positive
GSM1676125	Tumor biopsy (P-204)	Positive
GSM1676058	Tumor biopsy (P-232)	Positive
GSM1676030	Tumor biopsy (P-182)	Positive
GSM1676045	Tumor biopsy (P-202)	Positive
GSM1676205	Tumor biopsy (P-469)	Positive
GSM1676113	Tumor biopsy (P-368)	Positive
GSM1676077	Tumor biopsy (P-278)	Positive
GSM1676105	Tumor biopsy (P-349)	Positive
GSM1676024	Tumor biopsy (P-166)	Positive
GSM1676021	Tumor biopsy (P-149)	Positive
GSM1676063	Tumor biopsy (P-244)	Positive
GSM1676052	Tumor biopsy (P-220)	Positive
GSM1676132	Tumor biopsy (P-097)	Positive
GSM1676035	Tumor biopsy (P-191)	Positive
GSM1676149	Tumor biopsy (P-267)	Positive
GSM1675981	Tumor biopsy (P-018)	Positive
GSM1675993	Tumor biopsy (P-072)	Positive
GSM1676196	Tumor biopsy (P-455)	Positive

GSM1676038	Tumor biopsy (P-194)	Positive
GSM1676227	Tumor biopsy (P-509)	Positive
GSM1676014	Tumor biopsy (P-136)	Positive
GSM1676079	Tumor biopsy (P-281)	Positive
GSM1676195	Tumor biopsy (P-454)	Positive
GSM1676083	Tumor biopsy (P-288)	Positive
GSM1676009	Tumor biopsy (P-125)	Positive
GSM1676013	Tumor biopsy (P-133)	Positive
GSM1676034	Tumor biopsy (P-190)	Positive
GSM1676047	Tumor biopsy (P-208)	Positive
GSM1676165	Tumor biopsy (P-367)	Positive
GSM1676017	Tumor biopsy (P-142)	Positive
GSM1676218	Tumor biopsy (P-491)	Positive
GSM1676176	Tumor biopsy (P-411)	Positive
GSM1676091	Tumor biopsy (P-303)	Positive
GSM1676207	Tumor biopsy (P-474)	Positive
GSM1676223	Tumor biopsy (P-504)	Positive
GSM1676237	Tumor biopsy (P-526)	Positive
GSM1676221	Tumor biopsy (P-500)	Positive
GSM1676174	Tumor biopsy (P-409)	Positive
GSM1676071	Tumor biopsy (P-265)	Positive
GSM1676190	Tumor biopsy (P-434)	Positive
GSM1676199	Tumor biopsy (P-460)	Positive
GSM1676072	Tumor biopsy (P-270)	Positive
GSM1676081	Tumor biopsy (P-284)	Positive
GSM1676135	Tumor biopsy (P-201)	Positive
GSM1676111	Tumor biopsy (P-361)	Positive
GSM1675997	Tumor biopsy (P-080)	Positive
GSM1676230	Tumor biopsy (P-513)	Positive
GSM1676109	Tumor biopsy (P-355)	Positive
GSM1676096	Tumor biopsy (P-325)	Positive
GSM1676089	Tumor biopsy (P-294)	Positive
GSM1676153	Tumor biopsy (P-282)	Positive
GSM1676219	Tumor biopsy (P-493)	Positive
GSM1676010	Tumor biopsy (P-126)	Positive
GSM1676169	Tumor biopsy (P-397)	Positive
GSM1675978	Tumor biopsy (P-004)	Positive
GSM1676120	Tumor biopsy (P-389)	Positive
GSM1676076	Tumor biopsy (P-276)	Positive
GSM1676078	Tumor biopsy (P-279)	Positive
GSM1676016	Tumor biopsy (P-141)	Positive
GSM1676204	Tumor biopsy (P-468)	Positive
GSM1676186	Tumor biopsy (P-426)	Positive
GSM1676246	Tumor biopsy (P-538)	Positive

GSM1676142	Tumor biopsy (P-243)	Positive
GSM1676138	Tumor biopsy (P-211)	Positive
GSM1676220	Tumor biopsy (P-496)	Positive
GSM1676151	Tumor biopsy (P-277)	Positive
GSM1676114	Tumor biopsy (P-371)	Positive
GSM1676183	Tumor biopsy (P-421)	Positive
GSM1675999	Tumor biopsy (P-084)	Positive
GSM1676037	Tumor biopsy (P-193)	Positive
GSM1676222	Tumor biopsy (P-503)	Positive
GSM1676182	Tumor biopsy (P-420)	Positive
GSM1675998	Tumor biopsy (P-083)	Positive
GSM1676080	Tumor biopsy (P-283)	Positive
GSM1676145	Tumor biopsy (P-262)	Positive
GSM1676129	Tumor biopsy (P-041)	Positive
GSM1675987	Tumor biopsy (P-055)	Positive
GSM1676211	Tumor biopsy (P-481)	Positive
GSM1676070	Tumor biopsy (P-261)	Positive
GSM1676099	Tumor biopsy (P-332)	Positive
GSM1676085	Tumor biopsy (P-290)	Positive
GSM1676154	Tumor biopsy (P-297)	Positive
GSM1676025	Tumor biopsy (P-169)	Positive
GSM1676197	Tumor biopsy (P-456)	Positive
GSM1676235	Tumor biopsy (P-524)	Positive
GSM1676134	Tumor biopsy (P-179)	Positive
GSM1676202	Tumor biopsy (P-466)	Positive
GSM1676093	Tumor biopsy (P-318)	Positive
GSM1676171	Tumor biopsy (P-401)	Positive
GSM1676124	Tumor biopsy (P-134)	Positive
GSM1676181	Tumor biopsy (P-418)	Positive
GSM1676046	Tumor biopsy (P-203)	Positive
GSM1676107	Tumor biopsy (P-351)	Positive
GSM1676225	Tumor biopsy (P-506)	Positive
GSM1676108	Tumor biopsy (P-352)	Positive
GSM1676177	Tumor biopsy (P-412)	Positive
GSM1676012	Tumor biopsy (P-130)	Positive
GSM1676166	Tumor biopsy (P-370)	Positive
GSM1676023	Tumor biopsy (P-162)	Positive
GSM1676192	Tumor biopsy (P-442)	Positive
GSM1676208	Tumor biopsy (P-475)	Positive
GSM1676148	Tumor biopsy (P-266)	Positive
GSM1676162	Tumor biopsy (P-354)	Positive
GSM1676164	Tumor biopsy (P-366)	Positive
GSM1676214	Tumor biopsy (P-484)	Positive
GSM1676209	Tumor biopsy (P-478)	Positive

GSM1676112	Tumor biopsy (P-363)	Positive
GSM1675979	Tumor biopsy (P-005)	Positive
GSM1676167	Tumor biopsy (P-386)	Positive
GSM1676157	Tumor biopsy (P-310)	Positive
GSM1676184	Tumor biopsy (P-422)	Positive
GSM1676160	Tumor biopsy (P-337)	Positive
GSM1676065	Tumor biopsy (P-247)	Positive
GSM1676170	Tumor biopsy (P-399)	Positive
GSM1676031	Tumor biopsy (P-184)	Positive
GSM1676088	Tumor biopsy (P-293)	Positive
GSM1676062	Tumor biopsy (P-240)	Positive
GSM1676044	Tumor biopsy (P-200)	Positive
GSM1676172	Tumor biopsy (P-406)	Positive
GSM1676194	Tumor biopsy (P-445)	Positive
GSM1676042	Tumor biopsy (P-198)	Positive
GSM1676032	Tumor biopsy (P-188)	Positive
GSM1676128	Tumor biopsy (P-009)	Positive
GSM1676203	Tumor biopsy (P-467)	Positive
GSM1676020	Tumor biopsy (P-148)	Positive
GSM1675983	Tumor biopsy (P-028)	Positive
GSM1676064	Tumor biopsy (P-246)	Positive
GSM1676015	Tumor biopsy (P-138)	Positive
GSM1675980	Tumor biopsy (P-014)	Positive
GSM1676127	Tumor biopsy (P-008)	Positive
GSM1676242	Tumor biopsy (P-533)	Positive
GSM1676106	Tumor biopsy (P-350)	Positive
GSM1676043	Tumor biopsy (P-199)	Positive
GSM1676061	Tumor biopsy (P-239)	Positive
GSM1676087	Tumor biopsy (P-292)	Positive
GSM1676238	Tumor biopsy (P-527)	Positive
GSM1676122	Tumor biopsy (P-391)	Positive
GSM1676074	Tumor biopsy (P-274)	Positive
GSM1676051	Tumor biopsy (P-215)	Positive
GSM1676011	Tumor biopsy (P-129)	Positive
GSM1676147	Tumor biopsy (P-264)	Positive
GSM1676095	Tumor biopsy (P-322)	Positive
GSM1676094	Tumor biopsy (P-319)	Positive
GSM1676082	Tumor biopsy (P-285)	Positive
GSM1676027	Tumor biopsy (P-174)	Positive
GSM1676055	Tumor biopsy (P-227)	Positive
GSM1676200	Tumor biopsy (P-462)	Positive
GSM1676241	Tumor biopsy (P-532)	Positive
GSM1676232	Tumor biopsy (P-518)	Positive
GSM1676240	Tumor biopsy (P-531)	Positive

GSM1676117	Tumor biopsy (P-376)	Positive
GSM1676189	Tumor biopsy (P-433)	Positive
GSM1676100	Tumor biopsy (P-333)	Positive
GSM1676068	Tumor biopsy (P-257)	Positive
GSM1676059	Tumor biopsy (P-235)	Positive
GSM1676073	Tumor biopsy (P-272)	Positive
GSM1676066	Tumor biopsy (P-251)	Positive
GSM1675994	Tumor biopsy (P-073)	Positive
GSM1675996	Tumor biopsy (P-078)	Positive
GSM1676187	Tumor biopsy (P-427)	Positive
GSM1676175	Tumor biopsy (P-410)	Positive
GSM1676054	Tumor biopsy (P-222)	Positive
GSM1675977	Tumor biopsy (P-002)	Positive
GSM1676173	Tumor biopsy (P-408)	Positive
GSM1676007	Tumor biopsy (P-121)	Positive
GSM1676101	Tumor biopsy (P-340)	Positive
GSM1676000	Tumor biopsy (P-087)	Positive
GSM1675989	Tumor biopsy (P-062)	Positive
GSM1676198	Tumor biopsy (P-457)	Positive
GSM1676057	Tumor biopsy (P-231)	Positive
GSM1675988	Tumor biopsy (P-058)	Positive
GSM1676168	Tumor biopsy (P-395)	Positive
GSM1676159	Tumor biopsy (P-336)	Positive
GSM1676102	Tumor biopsy (P-343)	Positive
GSM1676231	Tumor biopsy (P-516)	Positive
GSM1675985	Tumor biopsy (P-042)	Positive
GSM1676233	Tumor biopsy (P-519)	Positive
GSM1676004	Tumor biopsy (P-101)	Positive
GSM1676001	Tumor biopsy (P-091)	Positive
GSM1675995	Tumor biopsy (P-074)	Positive
GSM1676188	Tumor biopsy (P-429)	Positive
GSM1676210	Tumor biopsy (P-479)	Positive
GSM1676139	Tumor biopsy (P-218)	Negative
GSM1676033	Tumor biopsy (P-189)	Negative
GSM1676075	Tumor biopsy (P-275)	Negative
GSM1676141	Tumor biopsy (P-229)	Negative
GSM1676115	Tumor biopsy (P-372)	Negative
GSM1676028	Tumor biopsy (P-177)	Negative
GSM1676216	Tumor biopsy (P-487)	Negative
GSM1676179	Tumor biopsy (P-416)	Negative
GSM1676212	Tumor biopsy (P-482)	Negative
GSM1676133	Tumor biopsy (P-163)	Negative
GSM1676226	Tumor biopsy (P-507)	Negative
GSM1676215	Tumor biopsy (P-485)	Negative

GSM1676185	Tumor biopsy (P-423)	Negative
GSM1675992	Tumor biopsy (P-068)	Negative
GSM1676002	Tumor biopsy (P-093)	Negative
GSM1676040	Tumor biopsy (P-196)	Negative
GSM1676146	Tumor biopsy (P-263)	Negative
GSM1676029	Tumor biopsy (P-180)	Negative
GSM1676136	Tumor biopsy (P-206)	Negative
GSM1676243	Tumor biopsy (P-535)	Negative
GSM1676098	Tumor biopsy (P-331)	Not Available
GSM1676110	Tumor biopsy (P-360)	Not Available
